# Fer-1 like family member 4 pseudogene: novel potential diagnostic and prognostic biomarker for cutaneous melanoma

**DOI:** 10.17179/excli2024-7719

**Published:** 2024-11-19

**Authors:** Tomasz Kolenda, Kacper Guglas, Maciej Stasiak, Paulina Poter, Joanna Kozlowska-Maslon, Piotr Bialas, Joanna Sobocinska, Marlena Janiczek-Polewska, Patrycja Mantaj, Anna Paszkowska, Zefiryn Cybulski, Anna Teresiak, Urszula Kazimierczak, Anna Przybyla, Andrzej Mackiewicz, Jacek Mackiewicz

**Affiliations:** 1 Department of Cancer Immunology, Chair of Medical Biotechnology, Poznan University of Medical Sciences, 8 Rokietnicka Street, 60-806 Poznan, Poland; 2 Department of Diagnostics and Cancer Immunology, Greater Poland Cancer Center, 15 Garbary Street, 61-866 Poznan, Poland; 3 Laboratory of Cancer Genetics, Greater Poland Cancer Center, 15 Garbary Street, 61-866 Poznan, Poland; 4 Greater Poland Cancer Center, Research and Implementation Unit, Garbary 15, 61-866 Poznan, Poland; 5 Postgraduate School of Molecular Medicine, 61 Zwirki i Wigury Street, 02-091 Medical University of Warsaw, Warsaw, Poland; 6 Department of Oncologic Pathology and Prophylaxis, Poznan University of Medical Sciences, Greater Poland Cancer Center, 15 Garbary Street, 61-866 Poznan, Poland; 7 Department of Pathology, Pomeranian Medical University, 1 Unii Lubelskiej Street, 71-242 Szczecin, Poland; 8 Faculty of Biology, Institute of Human Biology and Evolution, Adam Mickiewicz University, Uniwersytetu Poznańskiego 6, 61-614 Poznań, Poland; 9 Chair and Department of Cell Biology, Poznan University of Medical Sciences, 5D Rokietnicka, 60-806 Poznan, Poland; 10 Department of Clinical Oncology, Greater Poland Cancer Center, 61-866 Poznan, Poland; 11 Department of Electroradiology, Poznan University of Medical Sciences, 61-701 Poznan, Poland; 12 Greater Poland Cancer Center, Radiation Protection Department Greater Poland Cancer Center, 15 Garbary Street, 61-866 Poznan, Poland; 13 Faculty of Biology, Adam Mickiewicz University, Umultowska 89, 61-614 Poznan, Poland; 14 Department of Medical and Experimental Oncology, Institute of Oncology, Poznan University of Medical Sciences, Poznan, Poland

**Keywords:** FER1L4, C20orf124, pseudogene, non-coding RNA, lncRNA, miRNA, miR-514a-5p, miR-330-5p, miR-128-3p, TCGA, BRAF mutation, biomarkers

## Abstract

Cutaneous melanoma is the deadliest form of skin cancer. Despite advancements in treatment, many patients still face poor outcomes. A deeper understanding of the mechanisms involved in melanoma pathogenesis is crucial for improving diagnosis and therapy. Non-coding RNAs, with their extensive regulatory roles, show promise as diagnostic biomarkers. This study focuses on evaluating the *FER1L4* pseudogene and its potential role in melanoma. *FER1L4* expression was analyzed in normal melanocytes and melanoma cell lines using qRT-PCR. Additionally, TCGA data and online prediction tools were employed to correlate expression levels with clinicopathological features. The relationship between *FER1L4*, patient phenotypes, and immune responses was further explored using REACTOME, GSEA, and immune deconvolution analyses. *In vitro* analysis revealed significant upregulation of *FER1L4* in melanoma cells. Its expression levels were influenced by *BRAF* mutations and were markedly higher in metastatic compared to primary melanomas. Higher *FER1L4* expression was associated with improved patient survival. Furthermore, *miR-514a-5p*, *miR-330-5p*, and *miR-128-3p* were identified as interacting with *FER1L4*. Dysregulated genes involved in immune signaling pathways were also identified as potential miRNA targets. This is the first study to demonstrate the association of *FER1L4* with melanoma. Patients with elevated *FER1L4* levels exhibited distinct phenotypes, altered immunological profiles, and improved survival rates. These findings suggest that *FER1L4* could serve as a potential biomarker for melanoma.

## Background

Cutaneous melanoma derives from skin melanocytes, and it is responsible for over 75 % of skin cancer deaths worldwide (Corrie et al., 2014[9]) despite the introduction of educational programs, which based on the patients' declarations seem to be beneficial in the future in the reduction of skin cancer incidents (Dyzmann-Sroka, 2024[13]). The major melanoma risk factor is exposure to ultraviolet light (UV). However, the recent multifactorial risks theory is widely acknowledged. The UV causes damage to DNA structure, resulting in the development of melanoma, where the two most common *BRAF* gene mutations - *V600E* and *V600K* occur. These mutations are estimated as 80 % and 5-30 % of all *BRAF* mutations, respectively (Barbhaiya and Costenbader, 2014[3]; McArthur et al., 2014[49]). UV exposition results in the activation of the RAS/RAF/MEK/ERK mitogen-activated protein kinase (MAPK) signaling pathway, whose consequence is continuous cell proliferation and apoptosis inhibition (Garbe and Eigentler, 2018[19]). Melanoma appears in three subtypes: i) Superficial Spreading Melanoma (SSM), approximately 70 %, associated with exposure to UV, but may also derive from naevi; ii) Nodular Melanoma (NMM) - 5 % of cases, characterized by the rapid growth and high metastases formation; iii) Lentigo Maligna Melanoma (LMM) - accounts for 4-15 % cases and is correlated with long term UV exposure, and older age. Other subtypes occur very rarely (Rastrelli et al., 2014[54]).

Current standard treatment for melanoma includes both immunotherapy and targeted therapies. Immunotherapy focuses on blocking immune checkpoint inhibitors, such as cytotoxic T-cell antigen 4 (CTLA4), programmed cell death factor 1 (PD-1), PD-1 ligand, and lymphocyte-activation gene 3 (LAG3). In patients with *BRAF* mutations, targeted monotherapies using *BRAF* inhibitors or combination therapies are also employed. These often include BRAF and MEK1/2 inhibitors, where BRAF inhibition reduces cell proliferation and MEK1/2 inhibition halts MAPK signaling activation (Mackiewicz and Mackiewicz, 2018[47]). However, some melanoma cells exhibit primary or acquire secondary resistance to these treatments. Although the resistance mechanisms are well characterized, it is still difficult to overcome (Tangella et al., 2021[63]; Almeida et al., 2019[1]), and it causes metastases, such as brain metastases, which are difficult to treat (Lupattelli et al., 2022[43]). The pseudogenes have been recently described as regulatory transcripts. Similarly to long non-coding RNAs (lncRNAs), they are referred to as “junk DNA” and their biological role is still unclear. However, it has been demonstrated that some of the pseudogenes play an important role in carcinogenesis (Hu et al., 2018[23]). Pseudogenes can be divided into two groups - processed and unprocessed (Kovalenko and Patrushev, 2018[37]; Xu and Zhang, 2016[74]). The first group lacks introns since they originate from introns-free RNA and carry the poly-A tail (Tutar, 2012[65]). Most human pseudogenes are classified as processed ones (Kovalenko and Patrushev, 2018[37]). Pseudogenes are vital elements of the genome because they can positively or negatively regulate gene expression. As positive regulators, pseudogene transcripts can behave as miRNA “sponges” (An et al., 2017[2]). However, some of them can also act as negative regulators. They compete for RNA binding proteins (RBPs), which are important regulators of alternative splicing, polyadenylation, or the transport and localization of mRNA molecules (Hu et al., 2018[23]). If the competition for RBPs is successful, transcription of the parental gene is stopped (Hu et al., 2018[23]). This highlights the significant potential of pseudogenes as regulators and novel therapeutic targets.

Various RNA molecules have potential as diagnostic, prognostic, and predictive biomarkers, including those for metastasis or treatment response (Kolenda et al., 2017[30], 2019[34], 2020[28][29]; Łasińska et al., 2020[39]; Kozłowska-Masłoń et al., 2023[38]). Many studies have focused on miRNAs as potential biomarkers in melanoma (Mohammadpour et al., 2019[50]). However, the role of other non-coding RNA transcripts, such as lncRNAs and pseudogene transcripts, remains less understood and requires further investigation (Safa et al., 2020[55]; Guo et al., 2020[22]; Guglas et al., 2022[21]; Kolenda et al., 2023[31]).

This work is focused on the analysis of *FER1L4* (Fer-1 like family member 4 pseudogene; named also as *C20orf124*) transcript in human primary epidermal melanocytes and melanoma cell lines with different *BRAF* mutation status, as well as in melanoma patients based on the TCGA dataset. The report indicated the biological role of this pseudogene and pointed to its potential as a biomarker.

## Methods

### Cell lines

Human adult and neonatal primary epidermal melanocytes HEMa (ATCC® PCS-200-013) andHEMn (ATCC® PCS-200-012), respectively, and human melanoma cell lines MEWO, SKMEL28, WM115, WM266, WM9, and A549 were used for the study. Melanocyte cell lines were cultured according to the ATCC (American Type Culture Collection, Manassas, Virginia, USA) protocol. Melanoma cell lines were cultured as described previously (Czerwińska et al., 2020[10]). Information on the mutation status of the *BRAF* gene in cell lines was acquired from ATCC or DSMZ cell culture databases and verified as described previously (Mackiewicz-Wysocka et al., 2017[48]).

### qRT-PCR

Total RNA isolation and cDNA synthesis were performed as described previously (Czerwińska et al., 2020[10]). qRT-PCR was performed using 2x concentrated SYBR Green Master Mix (Roche) with specific primers to detect: *FER1L4* (ENSG00000088340) forward 5'-CCG TGT TGA GGT GCT GTT C-3' and reverse 5'-GGC AAG TCC ACT GTC AGA TG-3' designed as described previously (You et al., 2020[78]). All primers were verified using the NCBI BLAST tool. Real-time PCR was performed on a LightCycler 480 (Roche); the melting curve was created to discriminate between non-specific products. All real-time PCR data were analyzed by calculating the 2^-ΔCT^, normalizing against the *GAPDH* expression amplified using forward 5'-GCT CTC TGC TCC TCC TGT TC-3' and reverse 5'-ACG ACC AAA TCC GTT GAC TC-3' primers.

### Data sets

The TCGA data of selected transcripts were downloaded from XenaBrowser University of California, Santa Cruz, cohort: TCGA Melanoma (SKCM), cBioportal (Gao et al., 2013[17]), UALCAN (Chandrashekar et al., 2017) and ENCORI (Li et al., 2014[40]) databases. Full patient characteristics are presented in Supplementary Table 1. All data from XenaBrowser were available online, with unrestricted access, and patient consent is not required. The use of the data does not violate the rights of any person or any institution (Kolenda et., 2024[35]). 

### Clinical and pathological data analysis

Transcript expression levels were analyzed based on clinicopathological parameters, including sample type (primary vs. metastasis), cancer type (cutaneous melanoma vs. other), cancer localization (extremities, trunk, regional lymph node, head and neck, distant metastasis, cutaneous or subcutaneous tissue, or other), gender (female vs. male), age (<58 vs. >58), ulceration (absent vs. present), Clark level (I, II, III-IV, V), Breslow depth (<1, 1-2, 2.1-4, >4), mitotic rate (0-2, 2-3, >4), cancer stage (0, I+II, III+IV), M-stage (M0 vs. M1), and T-stage (T0, T1+T2, T3+T4). From a cohort of 472 patients, subgroups with high and low transcript expression were selected using quartile cutoffs: i) low (<25^th^ percentile) and ii) high (>75^th^ percentile). Progression-free survival (PFS) and overall survival (OS) were then assessed in these subgroups, similarly as described previously (Kolenda et al., 2023[32]).

### Phenotype analysis

Functional enrichment analysis and prediction of gene function were performed using Gene Set Enrichment Analysis (GSEA) software version 3.0 (http://www.gsea-msigdb.org/gsea/index.jsp). Melanoma patients were categorized into two groups with high and low expression of selected transcripts by the mean of the expression level. The input file contained expression data for 20,530 genes and 565 patients. One thousand gene set permutations were used for further analysis. Pathways (hallmark gene sets (H) and collection from MSigDB) with nominal p-value ≤ 0.05 and FDR ≤ 0.25 were considered significant (Grzechowiak et al., 2020[20]).

### miRNAs and their targets analysis

The molecular association between the *FER1L4* pseudogene transcript and miRNAs was investigated using the ENCORI database (Li et al., 2014[40]), focusing on 7mer-m8 interactions for base pairing. Selected miRNAs were further validated by identifying a significant negative correlation (R < -0.090, p < 0.05) between *FER1L4* expression and the miRNAs in melanoma patients, also using the ENCORI database. Clinicopathological data for these miRNAs were analyzed as outlined in section 2.4. Additionally, the relationship between miRNA expression levels and patient survival was evaluated by dividing patients into high and low expression subgroups based on the mean expression level of each miRNA, followed by statistical testing as described in section after next (Statistical analysis).

### Immune cell infiltration analysis

Immune and ESTIMATE Scores (Estimation of STromal and Immune cells in MAlignant Tumor tissues using Expression data) were downloaded from https://bioinformatics.mdanderson.org/estimate/disease.html and used to assess the infiltration of immune cells into tumor tissue and to infer tumor purity, as described previously (Kopczyńska et al., 2020[36]). Subpopulations of specific immune cells were estimated based on data presented by Thorsson et al. (2018[64]).

### Statistical analysis

All statistical analyses were conducted using GraphPad Prism (GraphPad, San Diego, CA, USA) and Statistica 13 (StatSoft, Poland). Depending on the normality of the data, assessed by the Shapiro-Wilk test, we applied the T-test, Mann-Whitney U test, or one-way ANOVA. Recurrence-free survival (RFS) and overall survival (OS) were analyzed using the log-rank (Mantel-Cox) test and the Gehan-Breslow-Wilcoxon test, respectively. All T-tests and ANOVA tests were two-tailed, with statistical significance set at p < 0.05.

## Results

### Expression of FER1L4 pseudogene is altered both in melanoma cell lines and melanoma patients

**Figure 1:**
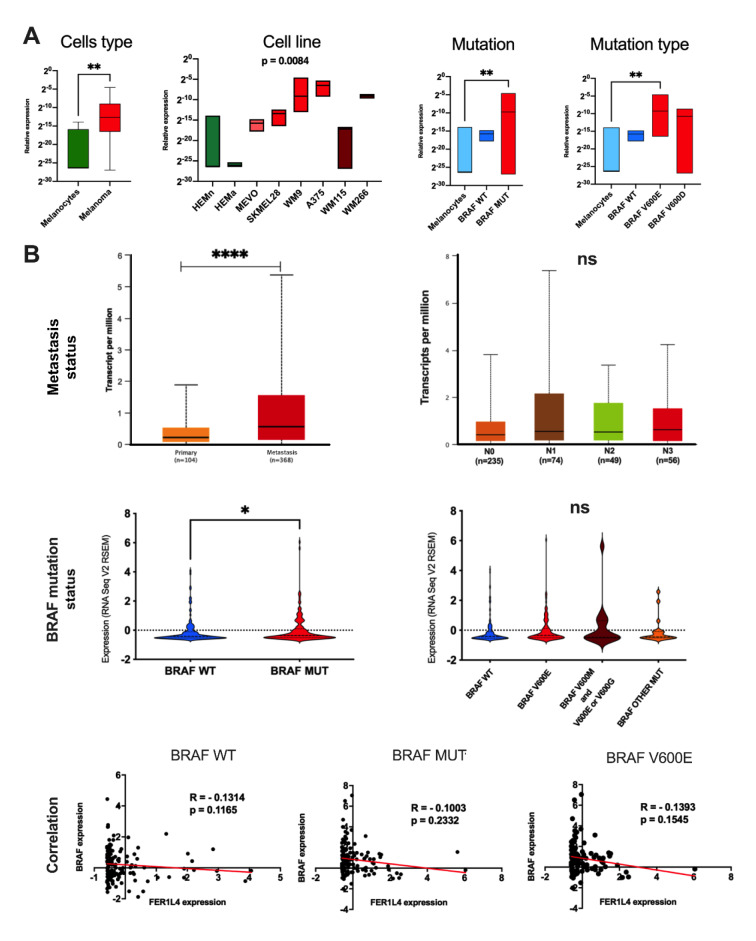
The *FER1L4* in melanoma cell lines and in TCGA patients: A) Expression levels in HEMn and HEMa melanocytes and melanoma cell lines depending on the cell type, cell line and *BRAF* status: *BRAF WT* (MEVO), *BRAF V600E* (SKMEL28, WM9 and A549) and *BRAF V600D *(WM115 and WM266) and B) Expression levels in melanoma patients from TCGA database depending on: primary or distant metastases samples and regional nodal metastases status and *BRAF* mutation status. Correlation of expression levels between *BRAF* and the *FER1L4* in melanoma *BRAF* wild type (WT), *BRAF* mutated (MUT) and *BRAF V600E* (c. 1799T>A) subgroups; Graphs for metastasis status were taken from UALCAN database, modified; T- test or One way ANOVA with Kruskal-Wallis test, Spearman correlation test, p < 0.05 considered as significant; ns - no significant, * p ≤ 0.05, ** p ≤ 0.01, **** p ≤ 0.0001

The expression levels of the *FER1L4* in normal melanocytes - HEMa and HEMn (*BRAF WT*) cell lines, and human melanoma cell lines MEWO (*BRAF WT*), SKMEL28, WM9, A375 (*BRAF V600E*), WM115, and WM266 (*BRAF V600D*) were measured using qRT-PCR. Expression of the *FER1L4* was significantly upregulated in melanoma cell lines compared to normal melanocytes (1.091 x 10^-5^ ± 1.090 x 10^-5 ^vs. 0.004901 ± 0.002654; p = 0.0034). Moreover, the expression level of the *FER1L4* differed depending on the specific cell line (p = 0.0086), and the only differences between melanocytes (*BRAF WT*) and melanoma cell lines displaying mutation *BRAF V600E* were noticed (1.091 x 10^-5^ ± 1.090 x 10^-5^ vs. 0.009174 ± 0.005027; p = 0.0077); (Figure 1A(Fig. 1)).

Then, the *FER1L4* pseudogene was examined in melanoma patients using TCGA data and showed significant differences in expression between primary and metastatic samples (0.229 vs. 0.599; p = 2.43 x 10^-6^). No significant differences in the *FER1L4* expression (p < 0.05) were observed between patients with distant and regional nodal metastases. The expression level of the *FER1L4* was slightly upregulated in *BRAF* mutated cells (-0.1170 ± 0.06932 vs. 0.02746 ± 0.08285; p = 0.0294), but the analysis of specific mutations did not support the observation (p = 0.1624). Finally, no correlation between the expression of *BRAF* and the *FER1L4* in the group of patients displaying *BRAF WT*, *BRAF MUT,* nor *BRAF V600E* was noticed (R = -0.1314, p = 0.1165; R = -0.1003, p = 0.2332; R = -0.1393, p = 0.1545, respectively); (Figure 1B(Fig. 1)).

### FER1L4 expression depends on clinicopathological parameters and its higher expression is associated with longer patients' survival

There were significant associations of the FER1L4 with Clark level, Breslow and T stage (p = 0.0462, p = 0.0022 and p = 0.0009, respectively). No significant differences (p > 0.05) were observed between the FER1L4 and gender, age, ulceration, mitotic rate, M stage, cancer type nor neoplasm disease stage (Table 1(Tab. 1)).

**Figure 2:**
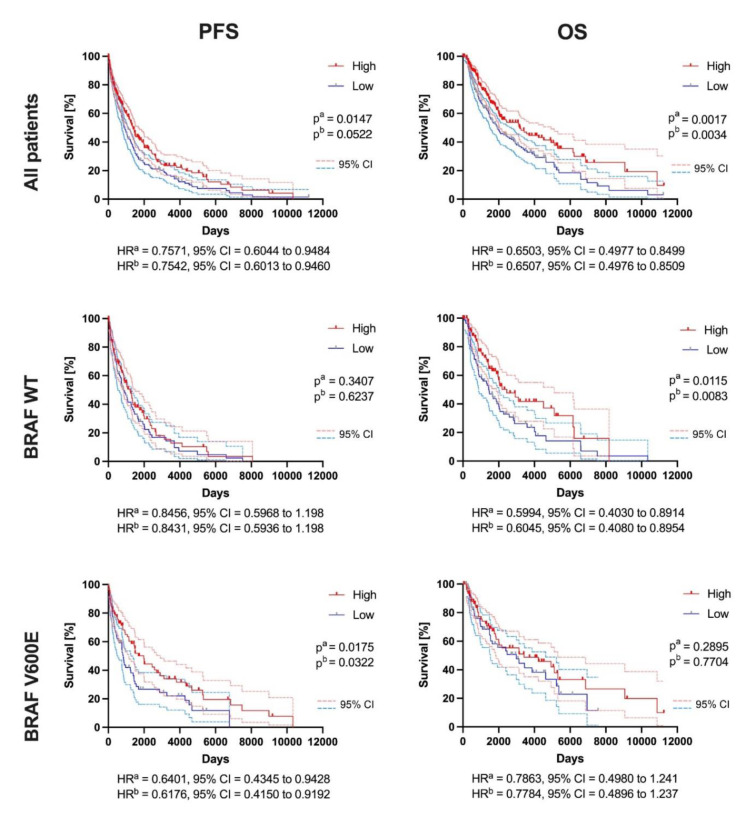
Progression-free survival (PFS) and overall survival (OS) of all melanoma patients, and in group of patients with *BRAF* gene wild type (WT) and mutation *BRAF V600E* depending on the *FER1L4* expression levels; low (blue solid lines) and high (red solid lines) subgroups represent patients depending on the expression level of specific gene, dashed light blue and red lines represents 95 % CI; low and high subgroups divided based on mean expression levels of the *FER1L4* in the specified group; p^a ^- Log-rank (Mantel-Cox) test, p^b ^- Gehan-Breslow-Wilcoxon test; HR - hazard ratio, CI - confidence interval; p < 0.05 considered as significant

For further analysis, melanoma patients were categorized into groups with high and low expression of the *FER1L4* and progression-free survival (PFS) as well as overall survival (OS) were assessed using Log-rank (p^a^) and Gehan-Breslow-Wilcoxon tests (p^b^) in the group of all melanoma patients and patients having non-mutated gene *BRAF* (wild type, WT) and displaying *V600E* mutation (c. 1799 T>A). Patients with higher expression levels of the *FER1L4* showed significantly longer PFS with median survival of 3.75 years (1372 days) in contrast to the low-expressing group of patients who had median survival estimated to 889 days *(*p^a^ = 0.0147, HR^a^ = 0.7571, 95 % CI = 0.6044 to 0.9484 and p^b^ = 0.0522, HR^b^ = 0.7542, 95 % CI = 0.6013 to 0.9460) as well as significantly longer OS with median survival 3195 vs. 1917 days (p^a^ = 0.0017, HR^a^ = 0.6503, 95 % CI = 0.4977 to 0.8499 and p^b^ = 0.0034, HR^b^ = 0.6507, 95 % CI = 0.4976 to 0.8509). In the case of only *BRAF WT* patients, no association between the *FER1L4* expression levels and PFS was observed (p > 0.05), but it was visible for OS. Patients with higher expression levels of that pseudogene had longer survival time with median estimated to 2454 vs. 1446 days (p^a^ = 0.0115, HR^a^ = 0.5994, 95 % CI = 0.4030 to 0.8914 and p^b^ = 0.0083, HR^b^ = 0.6045, 95 % CI = 0.4080 to 0.8954). Surprisingly, for patients with *V600E* mutation no association between *FER1L4* expression levels and OS was observed (*p* > 0.05). However, patients with higher expression levels of studied pseudogene had longer PFS with median survival estimated to 1981 vs. 829 days observed in the group with lower expression levels of the *FER1L4* (p^a^ = 0.0175, HR^a^ = 0.6401, 95 % CI = 0.4345 to 0.9428 and p^b^ = 0.0322, HR^b^ = 0.6176, 95 % CI = 0.4150 to 0.9192); (Figure 2(Fig. 2)).

The further analyses based on positive (R > 0.3) and negative (R < -0.3) Pearson correlations of the *FER1L4* with genes involved in important cellular processes were analyzed using the REACTOME pathway tool. For the *FER1L4*, 1072 positively correlated genes associated with the immune system, centrosomes maintenance, organelle biogenesis and maintenance and Rho GTPase were selected (p < 0.05). The analysis of 18 negatively regulated genes linked with the *FER1L4* indicated that they are involved in metabolisms, cellular transport, RNA metabolism and transcription (p < 0.05); (Supplementary Tables 2 and 3).

### FER1L4 is associated with several biological processes and signaling pathways in melanoma

**Figure 3:**
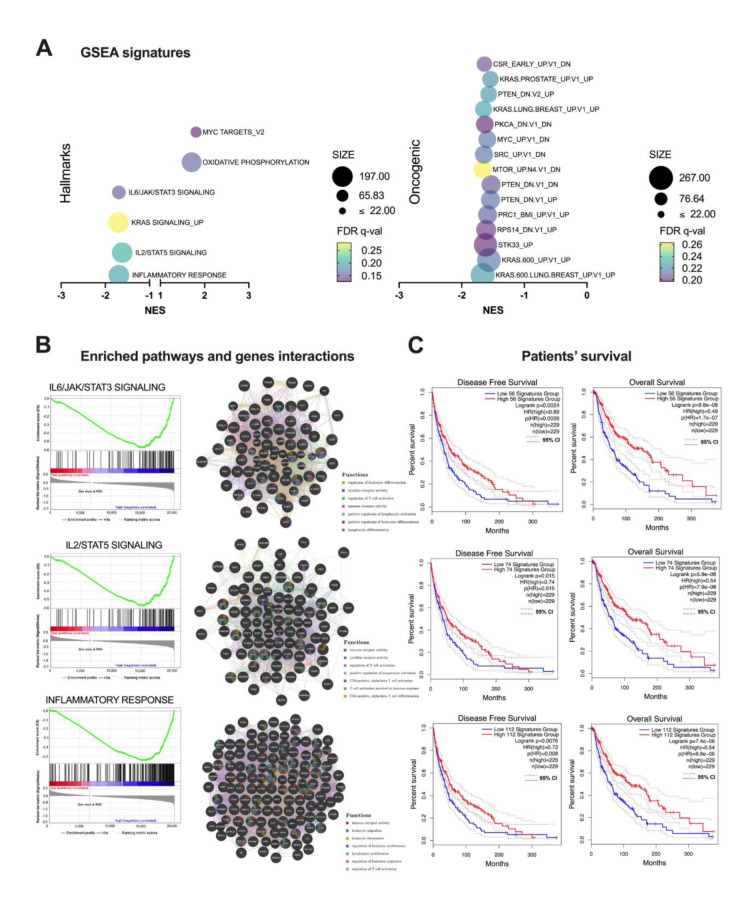
Biological role of the *FER1L4* in melanoma: A) GSEA results of TCGA melanoma patients analyzed in groups with low/high expression of the *FER1L4*; NES (normalized enrichment score), p-value (nominal p-value), FDR q-value (false discovery rate). Only results set with p ≤ 0.05 and FDR ≤ 0.25 are shown. B) Genes enriched with patients with higher levels of the *FER1L4* and their involvement in cellular processes based on the GENmania analysis tool and C) their association with disease-free survival and overall survival based on GEPIA2 database analysis (Log-rank (Mantel-Cox) test); low (blue solid lines) and high (red solid lines) subgroups represent patients depending on the expression level of specific group of genes, dashed light blue and red lines represents 95 % CI; HR - hazard ratio, CI - confidence interval; p < 0.05 considered as statistically significant

To further investigate the biological role of the *FER1L4*, patients were categorized into low and high expression subgroups of this pseudogene, and Gene Set Enrichment Analysis (GSEA) was performed. In patients with the *FER1L4* expression, there was notable enrichment in genes associated with MYC targets and oxidative phosphorylation. Conversely, patients with high *FER1L4* expression exhibited upregulation of genes related to inflammatory responses, including IL6-JAK-STAT3 signaling, IL2-STAT5 signaling, KRAS, JAK2_DN.V1_DN, MTOR_UP.N4.V1_DN, MYC_UP.V1_DN, and PTEN_DN.V2_UP, compared to the low-expression group. Detailed results are presented in Figure 3A(Fig. 3) and Supplementary Table 4.

Based on the GSEA analysis, *IL6/JAK/ STAT3*, *IL2/STAT5* signaling pathways, and inflammatory response enriched in a group of patients with higher levels of the *FER1L4* were selected for further analysis (Figure 3B(Fig. 3)). In the *IL6/JAK/STAT3* signaling pathway, 56 genes from this pathway were upregulated and connected with the regulation of leukocyte differentiation (11 genes), cytokine receptor activity (26 genes), regulation of T cell activation (11 genes), immune receptor activity (30 genes), positive regulation of lymphocyte activation (13 genes), positive regulation of leukocyte differentiation (8 genes), lymphocyte differentiation (13 genes) and other processes. *IL2/STAT5* signaling pathways were characterized by upregulation of 74 genes grouped in processes: immune receptor activity (11 genes), cytokine receptor activity (10 genes), regulation of T cell activation (14 genes), positive regulation of lymphocyte activation (13 genes), CD4-positive, alpha-beta T cell activation (4 genes) and other processes. The last enriched pathway observed in patients with higher levels of the *FER1L4* was inflammatory response, where 112 genes were changed and associated with: immune receptor activity (24 genes), leukocyte migration (28 genes), leukocyte chemotaxis (22 genes), regulation of leukocyte proliferation (19 genes), lymphocyte proliferation (18 genes), regulation of leukocyte migration (16 genes), regulation of T cell activation (18 genes) and other processes (Figure 3B(Fig. 3)). The list of genes enriched in specific pathways and their roles in various processes are listed in detail in Supplementary Tables 5 and 6.

Finally, the impact of gene expression levels from the *IL6/JAK/STAT3*, *IL2/STAT5* signaling pathways, and inflammatory responses on disease-free and overall survival was assessed (Supplementary Table 5). Patients with higher expression levels of genes from these pathways demonstrated significantly longer disease-free and overall survival compared to those with lower gene expression profiles (p = 0.0024 and p = 9.8 × 10^-8^; p = 0.015 and p = 5.9 × 10^-6^; p = 0.0076 and p = 7.4 × 10^-6^, respectively); (Figure 3C(Fig. 3)).

### FER1L4 pseudogene regulates miRNAs expression and their targets influencing on patients' survival

**Figure 4:**
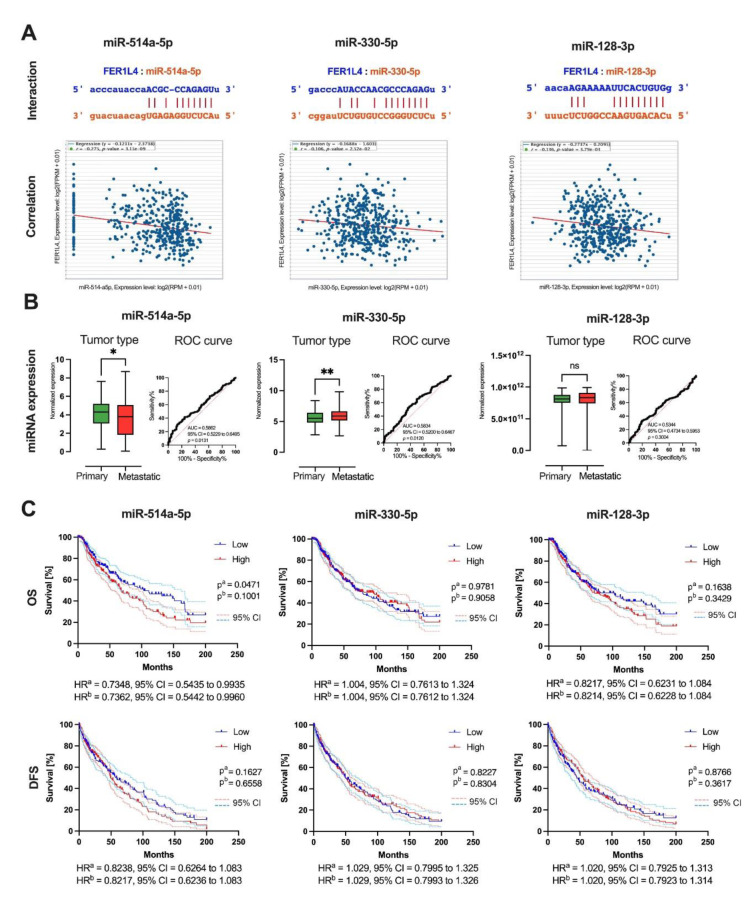
Molecular association of the *FER1L4* pseudogene transcript with miRNA: A) base pairing interaction with *miR-514a-5p*, *miR-330-5p* and *miR-128-3p* with the *FER1L4* and correlation between miRNA and pseudogene expression based on ENCORI database; B) expression levels of *miR-514a-5p*, *miR-330-5p* and *miR-128-3p* in patients with primary and metastatic melanoma with ROC curve; T-test or Mann-Whitney U test; AUC - area under ROC curve; C) association of *miR-514a-5p*, *miR-330-5p* and *miR-128-3p* expression levels with patients overall survival (OS) and disease-free survival (DFS); low (blue solid lines) and high (red solid lines) subgroups represent patients depending on the expression level of specific gene; p^a ^- Log-rank (Mantel-Cox) test, p^b^ - Gehan-Breslow-Wilcoxon test; HR - hazard ratio, CI - confidence interval; p < 0.05 considered as significant, ns - no significant, * p ≤ 0.05, ** p ≤ 0.01

The molecular association between the FER1L4 pseudogene transcript and miRNAs was analyzed using the ENCORI database. This analysis identified 24 miRNAs with potential base-pairing interactions with FER1L4 via 7mer-m8 interactions (Supplementary Table 7). Of these, only miR-514a-5p, miR-330-5p, and miR-128-3p showed a significant negative correlation with FER1L4 expression in melanoma patients (miR-514a-5p: R = -0.275, p = 3.15 × 10^-9^; miR-330-5p: R = -0.106, p = 0.0252; miR-128-3p: R = -0.136, p = 0.00379); (Figure 4A(Fig. 4)). The expression levels of these miRNAs were compared between primary and metastatic melanoma samples. miR-514a-5p was significantly downregulated in metastatic melanoma (mean ± SD: 3.475 ± 0.1159 vs. 4.070 ± 0.1755, p = 0.0129), while miR-330-5p was upregulated (mean ± SD: 5.920 ± 0.06212 vs. 5.581 ± 0.1081, p = 0.0098). No significant difference was observed for miR-128-3p (p = 0.3011). ROC curve analysis showed that miR-514a-5p had the highest ability to distinguish between metastatic and primary melanoma (AUC = 0.5862, 95 % CI = 0.5229 to 0.6495, p = 0.0131); (Figure 4B(Fig. 4)). Further analysis of the association between miR-514a-5p, miR-330-5p, and miR-128-3p expression levels and patient outcomes revealed that lower expression of miR-514a-5p was associated with longer overall survival (OS), (p = 0.0471, HR = 0.7348, 95 % CI = 0.5435 to 0.9935), while no significant association was found for miR-330-5p or miR-128-3p. No significant correlations were observed between miRNA expression levels and disease-free survival (DFS) for any of the miRNAs (p > 0.05).

**Figure 5:**
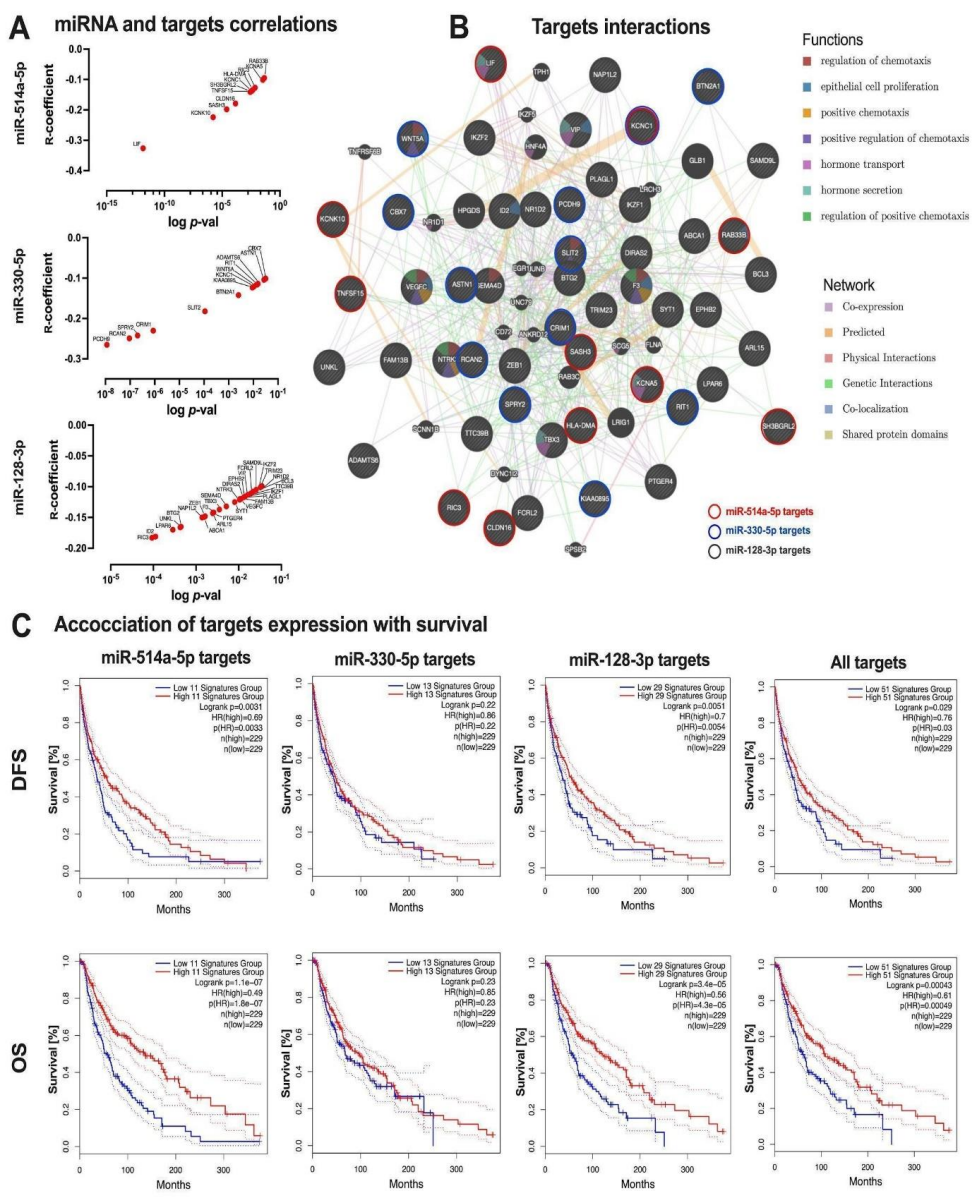
*miR-514a-5p*, *miR-330-5p* and *miR-128-3p* targets: A) correlation of mRNA targets with miRNAs based on ENCORI database, only significant (p < 0.05) genes were indicated; B) Net of interactions and functions between *miR-514a-5p*, *miR-330-5p* and *miR-128-3p* targets selected using the GeneMANIA tool; C) association with disease-free survival and overall survival of *miR-514a-5p*, *miR-330-5p*, *miR-128-3p* targets and all targets together based on GEPIA2 database analysis; (Log-rank (Mantel-Cox) test); low (blue solid lines) and high (red solid lines) subgroups represent patients depending on the expression level of specific group of genes, dashed light blue and red lines represents 95 % CI; HR - hazard ratio, CI - confidence interval; p < 0.05 considered as statistically significant

The targets of miRNAs interacting with the *FER1L4* pseudogene were identified using the miRNA target prediction database (miRDB) and cross-referenced with genes enriched in the GSEA analysis and verified using the ENCORI database. This analysis identified 11 genes targeted by *miR-514a-5p*, 13 genes targeted by *miR-330-5p*, and 29 genes targeted by *miR-128-3p* (Supplementary Table 8). These genes showed a negative correlation with their respective miRNAs in melanoma patients (R = -0.095 to -0.326, p < 0.05); (Figure 5A(Fig. 5)).

Interactions and functions of the targets of *miR-514a-5p*, *miR-330-5p*, and *miR-128-3p* were explored using the GeneMANIA tool. Among these targets, 76.14 % were co-expressed, 11.94 % shared the same predicted network, 4.13 % exhibited physical and genetic interactions, and 2.82 % were involved in co-localization. Identified functions included hormone secretion and transport, epithelial cell proliferation, and regulation of chemotaxis (Figure 5B(Fig. 5)). The association between the expression levels of these targets and patient survival was also assessed. Higher expression levels of* miR-514a-5p* and *miR-128-3p* targets were associated with longer disease-free survival (DFS) (p = 0.0031 and p = 0.0051, respectively) and overall survival (OS) (p = 1.1 × 10^-7^ and p = 3.4 × 10^-5^, respectively). No significant associations were found for *miR-330-5p* targets with DFS or OS (p = 0.220 and p = 0.230, respectively). Additionally, a combined analysis of all targets for these three miRNAs showed that patients with higher expression levels of these targets had longer DFS and OS (p = 0.029 and p = 0.00043, respectively) compared to those with lower expression levels (Figure 5B(Fig. 5)).

### Higher expression of FER1L4 pseudogene is associated with pro-survival phenotype in melanoma patients

**Figure 6:**
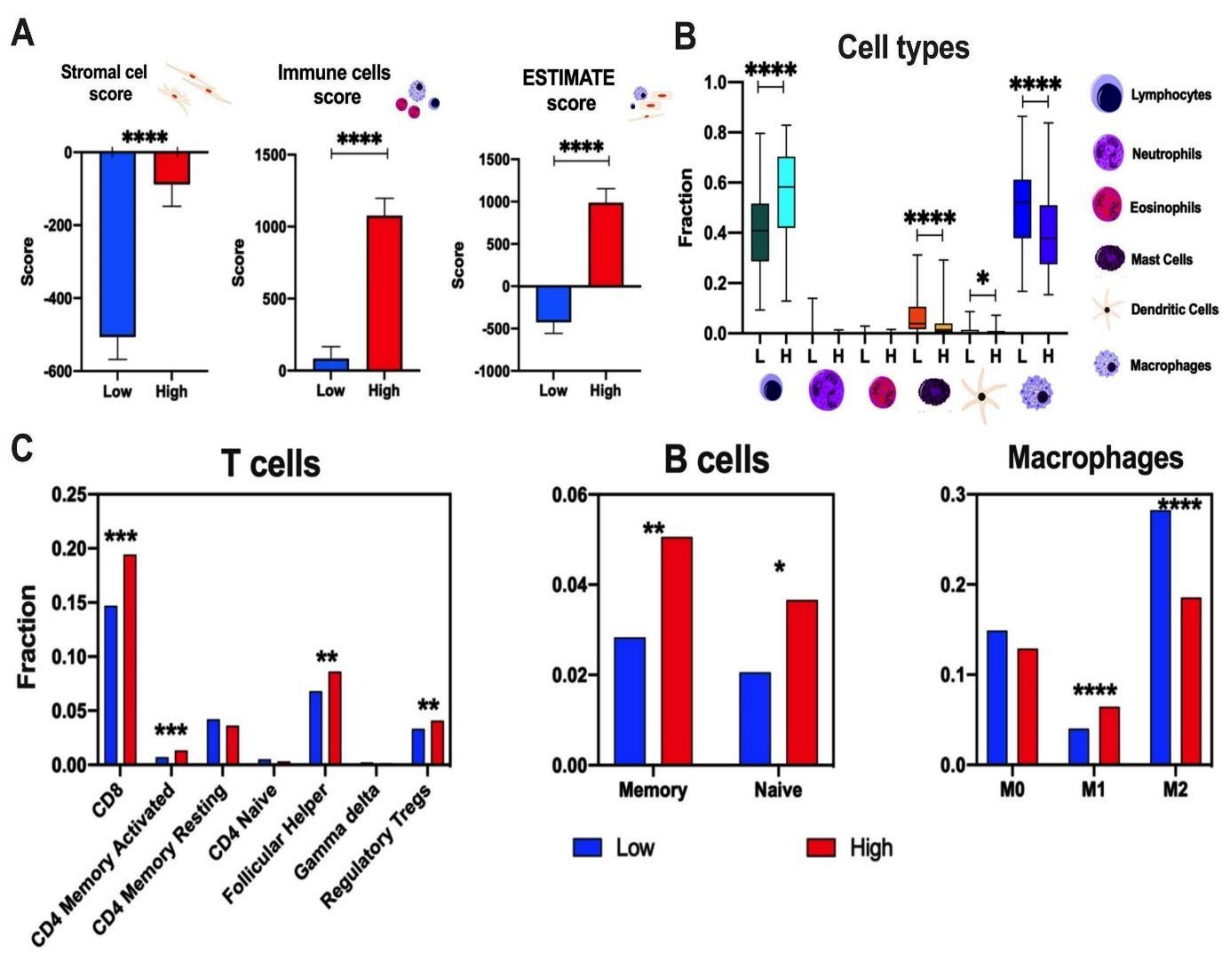
Immunological profile of TCGA melanoma patients depending on the expression level of the *FER1L4*: A) Assessment of ESTIMATE, immune cells and stromal cells scores; B) differences of lymphocytes, neutrophils, eosinophils, mast cells, dendritic cells and macrophages; and C) fraction of specific subpopulation of T cells, B cells and macrophages depending on the low and high expression level of analyzed transcripts; T-test, p < 0.05 considered as significant; * (p < 0.05), ** p ≤ 0.01, *** p ≤ 0.001, **** p ≤ 0.0001

Due to the association of the *FER1L4* pseudogene with the immune system, the abundance of immune cells, stromal cells, and cancer cells in patient samples was analyzed. Using the Immune and ESTIMATE Scores, significant differences between the *FER1L4* were found (p < 0.0001, p = 0.0483). Significantly higher immune score was observed in patients with higher expression of the *FER1L4* transcript (p < 0.0001). Moreover, a higher level of the *FER1L4* expression of stromal cells was indicated (p < 0.0001); (Figure 6A(Fig. 6)). Then, the differences in lymphocytes, neutrophils, eosinophils, mast cells, dendritic cells, and macrophages depending on the low and high expression levels of the analyzed pseudogene were assessed. In patients with higher *FER1L4* expression, a significantly higher fraction of lymphocytes was observed (p < 0.0001), along with a significantly lower fraction of mast cells (p < 0.0001), dendritic cells (*p* = 0.0210), and macrophages (p < 0.0001) (Figure 6B(Fig. 6)). Finally, the association between *FER1L4* expression levels and the fractions of specific subpopulations of T cells, B cells, and macrophages was analyzed. The patients with high expression levels of the *FER1L4* manifested higher infiltration of T cells CD8 (p = 0.0009), CD4 memory activated (*p* = 0.001), follicular helper (p = 0.0011) and regulatory Tregs (p = 0.0015), as well as higher memory and naive B cells (p = 0.0056 and p = 0.0107, respectively), and higher fraction of M1 and lower fraction of M2 macrophages subpopulations (p < 0.0001 and p < 0.0001, respectively); (Figure 6C(Fig. 6)).

## Discussion

It was noted that the *FER1L4* pseudogene levels were significantly upregulated in metastatic melanoma tissues, and its expression was associated with patient survival and immune profiles. Therefore, the *FER1L4* may serve as a valuable biomarker in melanoma.

To date, the *FER1L4* expression in melanoma has not been reported. Our study demonstrated that the *FER1L4* is upregulated in melanocytes and various melanoma cell lines, with expression levels correlated with *BRAF* mutations, particularly the *BRAF* V600E variant. These *in vitro* findings were corroborated in metastatic melanoma tissues from patients. The *FER1L4* (Fer-1 like family member 4 pseudogene) is a non-protein coding member of the Ferlin family. In addition to the non-coding transcript, the family comprises five protein-coding transcripts such as dysferlin, otoferlin, myoferlin, and Fer-1, like family member 5/6 (Bulankina and Thoms, 2020[4]). Ferlin proteins are involved in the secretory, endocytic and lysosomal pathways by their involvement in the function of membrane dynamics depending on calcium ions. These proteins possess multiple C2 domains (MC2D), which function as sensors and organizers of vesicular trafficking, signaling, lipid transfer and as enzymes for lipid modification. Changes in *Ferlins* are associated with human diseases, including cancers (Bulankina and Thoms, 2020[4]). You et al., based on the TCGA data and GEO described the *FER1L4* as an oncogene. Its higher expression level was associated with worse patients' outcomes. It was indicated that the pseudogene regulates important cellular processes such as DNA replication, cell proliferation and migration, mRNA polyadenylation, cell adhesion, and regulation of double strand break repair *via* homologous recombination. Moreover, they observed that the *FER1L4* may lead to the chemoresistance of cancer cells (You et al., 2020[78]). On the other hand, many studies indicated *FER1L4* as a tumor suppressor in prostate (Huo et al., 2020[27]), esophageal (Ma et al., 2018[45]), lung (Gao et al., 2019[18]; Ouyang et al., 2021[52]), gastric cancers (Xu et al., 2020[73]; Xia et al., 2015[71]), glioma (Xia et al., 2019[70]), osteosarcoma (Ye et al., 2019[77]; Fei et al., 2018[14]; Ma et al., 2019[44]), endometrial carcinoma (Qiao and Li, 2016[53]) and hepatocellular carcinoma (Wang et al., 2019[67]; Sun et al., 2019[62]).

The analysis of positively and negatively correlated genes and the gene set enrichment analysis demonstrated that depending on the *FER1L4* expression level, the crucial pathways involved in melanoma development; *IL6/JAK/STAT3*,* IL2/STAT5*, *KRAS*, *MTOR* or *PTEN*, were dysregulated (Tangella et al., 2021[63]; Almeida et al., 2019[1]; Yang et al., 2020[75]). Previous studies indicated that the *FER1L4* regulates processes such as proliferation, apoptosis, migration and invasion, and EMT process through *PI3K/AKT* signaling (Gao et al., 2019[18]; Ye et al., 2019[77]; Ma et al., 2019[44]; Wang et al., 2019[67]), also, with the involvement of *PTEN* and *p53* (Ouyang et al., 2021[52]; Xia et al., 2015[71]; Fei et al., 2018[14]; Qiao and Li, 2016[53]; Wang et al., 2019[67]), *Hippo-YAP* signaling pathways (Xu et al., 2020[73]) or *E2F1* (Xia et al., 2019[70]). Liu et al., indicated that upregulation of the *FER1L4* can overcome chemoresistance by changes in the *MAPK* signaling pathway in ovarian cancer cells (Liu et al., 2019[42]).

It should be strongly emphasized that knowledge about lncRNA and especially pseudogene transcripts in melanoma is limited. Guo et al., based on the TCGA data identified seven pseudogenes: *SRP9P1*, *RP4-800G7.1*, *CH17-264B6.3*, *C1DP1*, *MTND4P12*, *LDHAP3*, and *RP11-359E7.3* in melanoma tissue samples which are upregulated in melanoma. Among them only the expression of the *MTND4P12* was identified to be correlated with worse patients' survival. Further investigation indicated that the *MTND4P12* pseudogene acts as ceRNA and probably by regulating miR *let-7e-5p* regulates the expression of oncogene *AURKB* and finally influences cell phenotype. However, the authors did not analyze the association of those pseudogenes with clinical and pathological parameters; thus, it is difficult to conclude whether that observation was associated only with *MTND4P12* or depended on other factors such as metastatic status or treatment strategy (Guo et al., 2020[22]). Moreover, there are no studies on pseudogene transcripts alteration depending on the *BRAF* gene status in melanoma. Only a few studies were focused on the lncRNAs and *BRAF* gene and were based on melanoma tissue or cell lines. The most extensively studied non-coding RNAs in melanoma include *BRAF*-activated non-coding RNA (*BANCR*) and *RMEL-1*, *-2*, *-3* (Yu et al., 2017[79]; Sousa et al., 2010[57]). Liu et al., using the TCGA data, identified 438 differentially expressed lncRNAs between primary and metastatic melanoma patients. Among these, seven lncRNAs - *MIR205HG*, *LINC00200*, *LIFR-AS1*, *H19*, *MIAT*, A*C012640.1*, and *PLCH1-AS1 *- were proposed as potential prognostic signatures. These lncRNAs interact with mRNAs and miRNAs, forming a ceRNA network crucial for cancer progression (Liu et al., 2019[41]). In a similar study, Wang et al., identified 184 upregulated and 66 downregulated lncRNAs. Six of these lncRNAs - *AC068594.1*, *C7orf71*, *FAM41C*, *GPC5-AS1*, *MUC19*, and *LINC00402 *- were correlated with survival time in metastatic melanoma patients. However, this study did not determine if survival associations were solely due to these six lncRNAs or other factors (Wang et al., 2019[66][67][68]).

Another the TCGA-based study described six lncRNAs - *LINC01260*, *HCP5*, *PIGBOS1*, *RP11-247L20.4*, *CTA-292E10.6*, and *CTB-113P19.5*-that could classify patients into high-risk and low-risk groups with significantly different survival times. However, univariate and multivariate Cox regression models revealed that the lncRNA signature was influenced by age and cancer stage (Ma et al., 2017[46]). In contrast, Chen et al., validated only four lncRNAs - *HCP5*, *LIMD1-AS1*, *MIR155HG*, and *UNQ6494 *- as prognostic factors using the TCGA and GEO datasets. These lncRNAs demonstrated potential for risk stratification in melanoma patients, but it remains unclear if they are independent molecular biomarkers for melanoma (Chen et al., 2017[6]). Other studies identified the role of *HOTAIR*, *MALAT1*, *BANCR*, *ANRIL*, *SPRY‐IT1*, *SAMMSON*, *UCA1*, and *SLNCR1* in melanoma patients and cell lines. Those lncRNAs play a pivotal role in the regulation of migration, metastasis, proliferation, colony formation, and apoptosis of melanoma cells (Yu et al., 2018[80]).

In this study, we demonstrated that genes positively correlated with the *FER1L4* were associated with the following immunological processes: *RUNX3*-dependent regulation of immune response, cell migration, and *IL-2*,* -3*, *-5*, and *-10*, and *GM-CSF* signaling. Moreover, GSEA analysis indicated that patients with high expression of *FER1L4* represent phenotypes characteristic of the inflammatory response, *IL6-JAK-STAT3* signaling, and *IL2-STAT5* signaling. It is well known that *IL-2* is active in metastatic renal cell cancer and melanoma. It is linked to the activation of immune cells, as well as *JAK1/JAK3*, *STAT3*, *PI3K-AKT*, and *MAPK* signaling pathways (Conlon et al., 2019[8]). The activation of the STAT pathway was observed in melanoma patients with higher expression of the *FER1L4* compared to the patients with lower levels of this pseudogene. *GM-CSF*, *IL3*, and *IL-5* are the β common chain cytokines responsible for the regulation of varied inflammatory responses and activation of the *JAK/STAT* signaling pathway (Dougan et al., 2019[12]).

Huang et al., constructed a ceRNA network associated with bone metastases by profiling 104 primary melanomas and 8 bone metastatic melanomas from the TCGA. Their study also focused on identifying immune cell types in melanoma using RNA transcript analysis. They identified 8 pairs of lncRNA-miRNA interactions and 15 pairs of miRNA-mRNA connections. Notably, they discovered that lncRNA *AL118506.1* has significant prognostic value and is highly correlated with T follicular helper cells. It was also found to be negatively correlated with CD8+ T cells and M2 macrophages (Huang et al., 2019[24]). 

It is well known that pseudogenes can function as molecular sponges and regulate the level of miRNAs by binding their molecules, finally influencing the level of specific mRNA molecules (Stasiak et al., 2021[59]). In this study, we observed that *FER1L4* possesses bidding sites and is negatively correlated with three miRNAs named *miR-514a-5p*, *miR-330-5p*, and *miR-128-3p* in melanoma patients. Previously published studies described the regulation network between the *FER1L4* and *miR-18a-5p* (Ye et al., 2019[77]), *miR-372* (Xia et al., 2019[70]), *miR-874-3p* (Huang et al., 2020[25]) as well as *miR-106a-5p* (Yue et al., 2015[81]; Wu et al., 2017[69]; Xia et al., 2014[72]). However, Wang et al., based on the TCGA data of glioma patients, constructed a pseudogene-miRNA-mRNA regulatory network for the *FER1L4* in which *miR-514a-5p*, *miR-330-5p*, and *miR-128-3p* were included (Wang et al., 2019[66][67][68]). 

*miR-514a-5p* is a member of the *miR-506-514* cluster, which plays a role in melanocyte transformation and promotes melanoma growth (Streicher et al., 2012[60]). Notably, Stark et al., demonstrated that *miR-514a* is characteristic of melanoma and its overexpression inhibits *NF1* expression, ultimately supporting the survival of melanoma cells with the *BRAF* *V600E* mutation, even when treated with the BRAF inhibitor PLX4032 (Stark et al., 2015[58]). In contrast, *miR-330-5p* is downregulated in melanoma patients and cell lines. This miRNA is negatively correlated with the mitotic rate. Its upregulation has been shown to reduce melanoma cell proliferation, invasion, and migration abilities. Moreover, *miR-330-5p* directly targets *TYR* and *PDIA3*, which are responsible for melanogenesis and melanoma development (Su et al., 2016[61]). Sehati et al., also showed that *miR-330* had a suppressor ability and inhibits metastatic features of melanoma cells through downregulation of *CXCR4*, *VIM*, *MMP‐9*, and *MCAM* (*CD146*), and induces apoptosis through downregulation of *E2F1*, *AKT1*, and upregulation of caspase 3 (Sehati et al., 2020[56]). It should be noted that miR-330-5p targets *RUNX* family transcription factor 3 (*RUNX3*), which seems to be responsible for the regulation of *BCL2L11* (*BIM*) transcription, regulation of I-kappaB kinase/*NF-kappaB* signaling, and signaling by *NOTCH* in melanoma cells (Feng et al., 2022[15]). The last miRNA indicated in this study, *miR-128-3p*, was also described in melanoma. It is expressed on the low level and its higher expression had inhibitory effects on proliferation, migration, invasion, and induced apoptosis. It was shown that the direct target for *miR-128-3p* was *NTRK3* displaying oncogenic properties (Zhou et al., 2021[82]). As we demonstrated, the *FER1L4* pseudogene could regulate both oncogenic as well as suppressor miRNAs. The most interesting was the interaction of the *FER1L4*: *miR-514a-5p*:11-targets. Patients with higher expression of the *FER1L4* displayed higher expression of those eleven genes, which was associated with longer disease-free and overall survival. Five of them: *KCNA5*, *KCNA1*, *KCNA10* (D'Arcangelo et al 2019[11]), *LIF* (Humbert et al., 2015[26]), *RAB33B* (Ohbayashi and Fukuda, 2012[51]) were described previously in melanoma or other processes connected with pigmentation. It was not surprising that some of the identified targets for *miR-514a-5p* are connected with the immune system. One such target is *HLA-DMA*, a gene in the *HLA* class II complex responsible for antigen presentation and the initiation of immune responses. Chen et al., demonstrated that higher levels of *HLA* class II expression enhance anti-tumor immunity and inflammatory responses by presenting tumor antigens to immune cells. This is manifested by better patients' survival. Moreover, it could be used as a future biomarker or even a therapeutic target (Chen et al., 2019[7]). The next identified *miR-514a-5p* target was *TNFSF15*. Gadeyne et al., identified *TNFSF15* as highly overexpressed in *HLA-DR+* compared to *HLA-DR-* areas of melanoma samples. It is worth to be noted that *HLA-DR+* areas are characterized by anti-tumor immune cell infiltration with an enhanced antigen presentation in melanoma. Finally, it leads to an exhausted immune microenvironment. However, this phenomenon could be used to introduce the therapy based on anti-PD-1 inhibitors (Gadeyne et al., 2021[16]). 

It is well known that the cancer microenvironment has a vast impact on tumor development and response to treatment (Kolenda et al., 2018[33]). Due to this fact, we analyzed whether the expression levels of the *FER1L4* pseudogene were connected with infiltration of immune cells in patient samples. Our results indicated that patients with higher levels of the *FER1L4* present distinct immunological profiles. The *FER1L4* pseudogene was connected with a higher infiltration level of T cells: CD8, CD4 memory activated, and follicular helper, as well as higher memory and naive B cells, and a higher fraction of M1 and a lower fraction of M2 macrophage subpopulations. Yang et al., based on the TCGA data, calculated the immune and stromal scores using the ESTIMATE algorithm and the abundance of six infiltrating immune cells using the TIMER algorithm in melanoma patients. The authors observed that the prognosis of the patients with higher numbers of CD8+ T cells and neutrophils was better (Yang et al., 2020[76]). Furthermore, our results also indicated that patients with higher expression levels of the *FER1L4* had a larger fraction of CD8+ T cells and neutrophils, respectively. 

## Conclusions

Our results, for the first time, clearly showed the association of the *FER1L4* pseudogene with melanoma and with favorable immune profiles, as well as better patient survival. We observed that this pseudogene has an important role in the pathogenesis of melanoma by regulation of *miR-514a-5p*, *miR-330-5p*, as well as *miR-128-3p*, which influences melanoma and immune-related processes. Moreover, the *FER1L4* could be used as a potential biomarker and targeted therapy in the future.

## Declaration

### Acknowledgments

Acknowledgments to Greater Poland Cancer Center and Poznan University of Medical Sciences for supporting our work and providing a fully equipped laboratory to perform the necessary analyzes. This work was supported by the National Science Center, Poland, allocated based on decision no.: 2016/21/B/NZ7/01773 to JM.

### Authors' contributions

Authors' individual contributions: conceptualization: TK; methodology: TK; investigation: TK, KG, MS, PP, JK, PB, JS, APa, UK, APr, PM, MJP; data curation: TK, KG, MS, PP, JK; writing - original draft preparation: TK, KG; PB writing - review and editing: TK, JM, ZC, AM, UK, AT, PB; visualization: TK; supervision: JM, AM; funding acquisition: JM. TK's contribution to this paper is estimated to be 70 % of all authors' contributions. All authors read and approved the final manuscript.

### Funding

This work was supported by the National Science Center, Poland, allocated based on decision no.: 2016/21/B/NZ7/01773 to JM.

### Availability of data and materials

The datasets used during the current study are available online from XenaBrowser University of California, Santa Cruz, cohort: TCGA Melanoma (SKCM), cBioportal, UALCAN and ENCORI databases, or from the corresponding author on reasonable request.

### Ethics approval

All data is available online, access is unrestricted and does not require patient's consent or other permissions. The use of the data does not violate the rights of any person or any institution. 

### Consent for publication

All authors read and approved the final manuscript.

### Competing interests

The authors declare that there is no conflict of interest regarding the publication of this paper. The use of the data does not violate the rights of any person or any institution.

## Supplementary Material

Supplementary information

## Figures and Tables

**Table 1:**
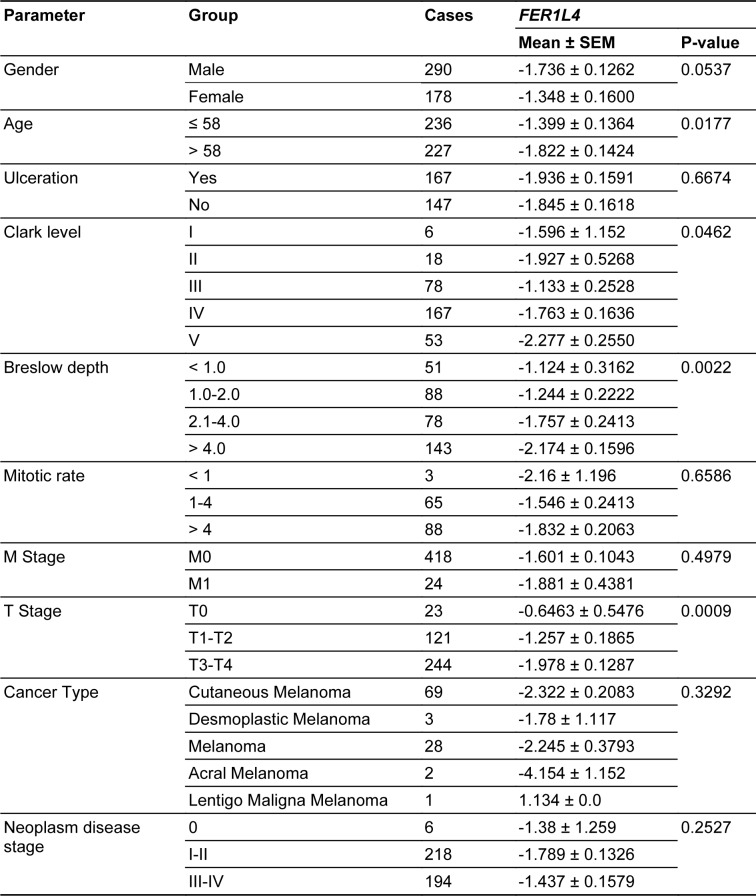
The expression levels of the *FER1L4* versus clinicopathological parameters characterizing the TCGA melanoma patients; p < 0.05 considered as significant
