# Supplementary material for: Fer-1 like family member 4 pseudogene: novel potential diagnostic and prognostic biomarker for cutaneous melanoma
Source: EXCLI J. 2024 Nov 19;23:1375–96. doi: 10.17179/excli2024-7719 (PMC11701299; doi:10.17179/excli2024-7719)
Supplement: Supplementary information [file EXCLI-23-1375-s-001.pdf]

EXCLI Journal 2024;23:1375-1396 – ISSN 1611-2156
Received: August 08, 2024, accepted: November 06, 2024, published: November 19, 2024

Supplementary information to:

Original article:

FER-1 LIKE FAMILY MEMBER 4 PSEUDOGENE: NOVEL POTENTIAL DIAGNOSTIC AND PROGNOSTIC BIOMARKER FOR CUTANEOUS MELANOMA

Tomasz Kolenda[1,2,3,8], Kacper Guglas[3,4,8], Maciej Stasiak[3,8], Paulina Poter[5,6,8], Joanna Kozłowska-Masłoń[3,8,9], Piotr Białas[10], Joanna Sobocińska[3,8], Marlena Janiczek-Polewska[13,14], Patrycja Mantaj[12], Anna Paszkowska[3,8,11], Zefiryn Cybulski[8], Anna Teresiak[3,8], Urszula Kazimierczak[1,2], Anna Przybyła[1,2], Andrzej Mackiewicz[1,2], Jacek Mackiewicz[2,7]

1 Department of Cancer Immunology, Chair of Medical Biotechnology, Poznan University of Medical Sciences, 8 Rokietnicka Street, 60-806 Poznan, Poland
2 Department of Diagnostics and Cancer Immunology, Greater Poland Cancer Center, 15 Garbary Street, 61-866 Poznan, Poland
3 Laboratory of Cancer Genetics, Greater Poland Cancer Center, 15 Garbary Street, 61-866 Poznan, Poland
4 Postgraduate School of Molecular Medicine, 61 Zwirki i Wigury Street, 02-091 Medical University of Warsaw, Warsaw, Poland
5 Department of Oncologic Pathology and Prophylaxis, Poznan University of Medical Sciences, Greater Poland Cancer Center, 15 Garbary Street, 61-866 Poznan, Poland
6 Department of Pathology, Pomeranian Medical University, 1 Unii Lubelskiej Street, 71-242 Szczecin, Poland
7 Department of Medical and Experimental Oncology, Institute of Oncology, Poznan University of Medical Sciences, Poznan, Poland
8 Greater Poland Cancer Center, Research and Implementation Unit, Garbary 15, 61-866 Poznan, Poland
9 Faculty of Biology, Institute of Human Biology and Evolution, Adam Mickiewicz University, Uniwersytetu Poznańskiego 6, 61-614 Poznań, Poland
10 Chair and Department of Cell Biology, Poznan University of Medical Sciences, 5D Rokietnicka, 60-806 Poznan, Poland
11 Faculty of Biology, Adam Mickiewicz University, Umultowska 89, 61-614 Poznan, Poland
12 Greater Poland Cancer Center, Radiation Protection Department Greater Poland Cancer Center, 15 Garbary Street, 61-866 Poznan, Poland
13 Department of Clinical Oncology, Greater Poland Cancer Center, 61-866 Poznan, Poland
14 Department of Electroradiology, Poznan University of Medical Sciences, 61-701 Poznan, Poland

* **Corresponding author:** Tomasz Kolenda, Greater Poland Cancer Center, Research and Implementation Unit, Garbary 15, 61-866 Poznan, Poland. E-mail: tomasz.kolenda@wco.pl

https://dx.doi.org/10.17179/excli2024-7719

This is an Open Access article distributed under the terms of the Creative Commons Attribution License (http://creativecommons.org/licenses/by/4.0/).

EXCLI Journal 2024;23:1375-1396 – ISSN 1611-2156
Received: August 08, 2024, accepted: November 06, 2024, published: November 19, 2024

Supplementary Table 1: Patient characteristics based on data taken from the XenaBrowser database and taken to the study

Parameter	Group	Cases
Gender	Male	292
	Female	178
	Missing data	0
Age	≤58	237
	>58	228
	Missing data	5
Ulceration	Yes	168
	No	147
	Missing data	155
Clark level	I	6
	II	18
	III	78
	IV	167
	V	54
	Missing data	147
Breslow depth	<1.0	51
	1.0-2.0	88
	2.1-4.0	78
	>4.0	144
	Missing data	109
Mitotic rate	<1	4
	1-4	66
	>4	88
	Missing data	312
M Stage	M0	419
	M1	24
	Missing data	27
T Stage	T0	23
	T1-T2	121
	T3-T4	245
	Missing data	81
Cancer Type	Cutaneous Melanoma	69
	Desmoplastic Melanoma	3
	Melanoma	28
	Acral Melanoma	2
	Lentigo Maligna Melanoma	1
	Missing data	365
Neoplasm disease stage	0	6

EXCLI Journal 2024;23:1375-1396 – ISSN 1611-2156
Received: August 08, 2024, accepted: November 06, 2024, published: November 19, 2024

	I-II	218
	III-IV	195
	Missing data	51
BRAF status	Mutated	143
	Wild type	144
	Missing data	181
Tumor tissue site	Distant Metastasis	64
	Primary Tumor	105
	Regional Cutaneous or Sub-cutaneous Tissue (includes satellite and in-transit me-tastasis)	74
	Regional Lymph Node	224
	Missing data	3
Sample type	Additional Metastatic	1
	Metastatic	364
	Primary Tumor	105
Radiation therapy	No	419
	Yes	50
	Missing data	1
Prior systemic therapy type	Chemotherapy	1
	Immunotherapy/Vaccine	3
	Interferon	31
	Other	1
	Missing data	434
New tumor event after initial treatment	No	213
	Yes	251
	Missing data	6
New tumor metastasis anatomic site	Brain	16
	Lung	7
	Bone	1
	Liver	2
	Multiple	23
	Other (single)	7
	Missing data	414
Additional pharmaceutical therapy	No	31
	Yes	23
	No next Yes	9
	Yes next No	3
	Missing data	404
Additional radiation therapy	No	49
	Yes	10

EXCLI Journal 2024;23:1375-1396 – ISSN 1611-2156
Received: August 08, 2024, accepted: November 06, 2024, published: November 19, 2024

	No next Yes	1
	Yes next No	7
	Missing data	403

EXCLI Journal 2024;23:1375-1396 – ISSN 1611-2156
Received: August 08, 2024, accepted: November 06, 2024, published: November 19, 2024

Supplementary Table 2: List of genes positively and negatively correlated with *FER1L4* in melanoma patients based on CbioPortal. Only genes with R-Pearson <-0.3 and >0.3 were indicated.

Positively correlated with FER1L4																					
**Gene**	**R**	**Gene**	**R**	**Gene**	**R**	**Gene**	**R**	**Gene**	**R**	**Gene**	**R**	**Gene**	**R**	**Gene**	**R**	**Gene**	**R**	**Gene**	**R**	**Gene**	**R**
IGJ	0.61	RCSD1	0.45	MGC16142	0.41	PRDM1	0.39	IFT80	0.37	SRGAP3	0.35	ZSCAN16	0.34	GOLGB1	0.33	PPIL3	0.32	C20orf194	0.31	CD247	0.3
PIM2	0.6	YPEL1	0.45	TAS2R14	0.41	ZFP62	0.39	CCDC45	0.37	ZNF557	0.35	SLC6A6	0.34	PRX	0.33	WDR90	0.32	DCHS1	0.31	AKAP8	0.3
FAM46C	0.6	PLEKHM1P	0.45	ZNF700	0.41	ZNF169	0.39	SNAI3	0.37	TIFA	0.35	C1orf156	0.34	STAM2	0.33	ADAT2	0.32	PMS1	0.31	HMGB1	0.3
KIAA1009	0.59	LRIG1	0.45	TRIM59	0.41	NCRNA00201	0.39	OGT	0.37	ZNF136	0.35	CASP8AP2	0.34	FLJ36031	0.33	IL1RAP	0.32	ODF3B	0.31	NAA16	0.3
MGC29506	0.59	VAV1	0.45	LOC100131434	0.41	ITSN2	0.39	GPATCH2	0.37	C2orf67	0.35	KIAA0182	0.34	PHF11	0.33	P2RY13	0.32	TRAM1L1	0.31	C10orf4	0.3
LOC100132287	0.59	SPP1	0.44	SP140L	0.41	SASH3	0.39	NBPF16	0.37	CEP290	0.35	GGTA1	0.34	AKIRIN2	0.33	FMNL2	0.32	ZNF251	0.31	ANKRD34A	0.3
MIAT	0.58	LOC100129534	0.44	NKTR	0.41	KHDRBS3	0.39	DDHD1	0.37	CXCL2	0.35	RGNEF	0.34	C8orf48	0.33	ATP8B1	0.32	MEF2C	0.31	RANBP6	0.3
C1RL	0.57	SLC26A10	0.44	NCKAP1L	0.41	APBB1IP	0.39	LCAT	0.37	MAP3K4	0.35	ANO8	0.34	C3orf47	0.33	CD6	0.32	PHC3	0.31	CNOT6	0.3
KIAA1407	0.57	SRGN	0.44	ZBED5	0.41	VMO1	0.38	TGFB2	0.37	KIAA1143	0.35	KAT2B	0.34	TSGA10	0.33	CARD9	0.32	FCGR1B	0.31	CD79B	0.3
CMAH	0.57	KLHL3	0.44	PRKX	0.41	ZNF594	0.38	MDM1	0.37	KCTD18	0.35	C3orf71	0.34	PVRIG	0.33	GCFC1	0.32	TAF1	0.31	BEND5	0.3
PRRT2	0.57	C5orf56	0.44	DOPEY1	0.4	CCDC104	0.38	PCDHB7	0.37	CCNL2	0.35	MAP1D	0.34	AP4B1	0.33	NR1D2	0.32	TNRC6C	0.31	HSD17B7P2	0.3
DNAH1	0.56	SP140	0.44	BTG2	0.4	NPFF	0.38	WDR35	0.37	C1orf27	0.35	P2RY1	0.34	NIPSNAP3A	0.33	CDH11	0.32	MTR	0.31	MED7	0.3
LOC100133331	0.56	SLC26A2	0.44	ZNF709	0.4	ZMYND17	0.38	NRCAM	0.37	ANKRA2	0.35	RGPD4	0.34	ANKRD12	0.33	ALPK1	0.32	PPP1R12A	0.31	PDGFA	0.3
MTMR11	0.54	APOBEC3G	0.44	AGER	0.4	LIMS1	0.38	ZFP14	0.37	CD33	0.35	CEP97	0.34	UBA5	0.33	CDRT4	0.32	ESR1	0.31	PDE7A	0.3
RPL32P3	0.54	IFT57	0.44	MCL1	0.4	C1orf26	0.38	NR2C2	0.37	SCAI	0.35	TBC1D10C	0.34	MTRF1	0.33	ZNF84	0.32	ZNF767	0.31	FCGR1A	0.3
C17orf28	0.53	INE1	0.44	MTX3	0.4	C10orf137	0.38	TRDMT1	0.37	LIME1	0.35	CCR5	0.34	TET3	0.33	ZNF720	0.32	LOC653501	0.31	AKAP11	0.3
ST8SIA4	0.53	KTELC1	0.44	C6orf204	0.4	SDCCAG8	0.38	CDK3	0.37	ZNF791	0.35	LZTFL1	0.34	SPPL2B	0.33	LEKR1	0.32	PY-ROXD1	0.31	STX2	0.3
C1R	0.53	LOC400927	0.44	BTN3A1	0.4	THOC1	0.38	SETD2	0.37	FAM193B	0.35	SLA	0.34	NCRNA00107	0.33	HECA	0.32	AKAP7	0.31	LIG4	0.3
ADAM6	0.53	CEP120	0.44	BCL6	0.4	CMTM6	0.38	AFAP1L2	0.37	FCHO2	0.35	LRP5L	0.34	ABI2	0.33	RAC2	0.32	LMLN	0.31	CCDC93	0.3
LOC91316	0.53	ARHGAP25	0.44	C21orf7	0.4	C16orf52	0.38	LOC728190	0.37	LOC96610	0.35	TRIP10	0.34	CRIPAK	0.33	ZNF670	0.32	CRYBG3	0.31	ITGA4	0.3
TM6SF1	0.52	BIN2	0.44	CD46	0.4	BCAT1	0.38	LOC285359	0.37	DOCK11	0.35	RBM5	0.34	ZNF596	0.33	ALX3	0.32	KCNJ14	0.31	ADM2	0.3
SAMSN1	0.52	NAMPT	0.44	HEXDC	0.4	CDC40	0.38	IL2RB	0.37	TTC13	0.35	MAMDC4	0.34	CDKN1B	0.33	SETD5	0.32	RPIA	0.31	ZC3H11A	0.3

EXCLI Journal 2024;23:1375-1396 – ISSN 1611-2156
Received: August 08, 2024, accepted: November 06, 2024, published: November 19, 2024

CD48	0.52	NCRNA00095	0.44	CXCL9	0.4	LAMB2L	0.38	CAMK2G	0.36	UNKL	0.35	MIA3	0.34	USPL1	0.33	CCNT2	0.32	GCNT1	0.31	DDHD2	0.3
DERL3	0.52	LOC375190	0.44	CLK4	0.4	ANKRD10	0.38	CSNK1G3	0.36	FPR1	0.35	NCF1B	0.34	SLFN13	0.33	FCGR1C	0.32	BCKDHB	0.31	QKI	0.3
EAF2	0.52	LOC100190986	0.44	GORAB	0.4	KCTD6	0.38	TAS2R20	0.36	XRN1	0.35	DLG3	0.34	COL18A1	0.32	KIAA0907	0.32	ATR	0.31	LOC401052	0.3
CD38	0.51	TCTE3	0.43	IL10RA	0.4	ZNF627	0.38	RWDD2A	0.36	NCOA7	0.35	FAM35B2	0.34	RICTOR	0.32	CSF1R	0.32	ZNF70	0.31	ATP11B	0.3
WDR52	0.51	KPNA5	0.43	LOC221442	0.4	MSR1	0.38	ZNF25	0.36	MAP4K1	0.35	NEK11	0.34	SHANK3	0.32	AKNA	0.32	MCOLN2	0.31	CRAMP1L	0.3
LOC100132215	0.51	IRAK1BP1	0.43	ARID4B	0.4	KIAA1370	0.38	SH3BGRL2	0.36	PTAR1	0.35	GKAP1	0.34	NPHP3	0.32	ETV7	0.32	VEGFA	0.31	CCDC157	0.3
PCDHB14	0.51	PTPN7	0.43	AKD1	0.4	C5orf45	0.38	CD86	0.36	ZNF512	0.35	HCK	0.34	PCNP	0.32	RASAL3	0.32	LOC344595	0.31	KIAA1712	0.3
SFRS18	0.51	MAPK8IP3	0.43	CD69	0.4	CEP70	0.38	NLRC5	0.36	TP53INP1	0.35	ARGLU1	0.34	OBFC1	0.32	TNRC6A	0.32	MYSM1	0.31	PTPRCAP	0.3
MEI1	0.51	LAT2	0.43	ZNF33A	0.4	AGAP2	0.38	MGAT4A	0.36	ZMYND15	0.35	CEP110	0.34	CWC22	0.32	DYNC2LI1	0.32	HSBP1	0.31	POLI	0.3
C3	0.5	INPP5D	0.43	EPN2	0.4	CXorf23	0.38	MGEA5	0.36	ACVR2A	0.35	CNOT8	0.34	MGAT3	0.32	NPHP1	0.32	ITGAL	0.31	ARL4C	0.3
PTPRC	0.5	FAM55C	0.43	LOC339047	0.4	APOBEC3D	0.38	NFIA	0.36	PLA2G6	0.35	C6orf150	0.34	N4BP2L1	0.32	STEAP3	0.32	LYPLAL1	0.31	CBR4	0.3
CD79A	0.5	NFE2L3	0.43	FAM95B1	0.4	LOC730101	0.38	NEIL1	0.36	ARHGAP12	0.35	MSL2	0.34	ZNF230	0.32	REC8	0.32	HMGN3	0.31	GPR126	0.3
ZNF37B	0.5	GBP5	0.43	SCARNA17	0.4	FAM35B	0.38	SVIL	0.36	AMIGO3	0.35	SELL	0.33	RHPN1	0.32	CASP8	0.32	FAM190B	0.31	DENND4A	0.3
TRIM52	0.5	ZNF518A	0.43	CMTM8	0.4	TIA1	0.38	APOBEC3F	0.36	LOC440944	0.35	LOC338758	0.33	TNFRSF1B	0.32	CLINT1	0.32	PSTPIP2	0.31	GABPA	0.3
LOC100129637	0.5	MDM4	0.43	HECTD2	0.4	RC3H1	0.38	THBS1	0.36	TTC25	0.35	TMED7	0.33	GIN1	0.32	ABAT	0.32	INSIG2	0.31	LYN	0.3
TTLL3	0.5	CHKB-CPT1B	0.43	XRCC4	0.4	ZMYND11	0.38	NCRNA00115	0.36	ZNF101	0.35	DDX50	0.33	MPP3	0.32	KRCC1	0.31	GLIPR1	0.31	PION	0.3
MEF2B	0.5	GNRH1	0.43	AGAP4	0.4	UFM1	0.38	SOCS5	0.36	ZNF708	0.35	CD28	0.33	CDC42SE2	0.32	CALCOCO2	0.31	IFT20	0.31	CCDC82	0.3
GAB3	0.5	P2RY8	0.43	ERRFI1	0.4	SH3YL1	0.38	P2RY11	0.36	CROCCL2	0.35	NME5	0.33	KIF21B	0.32	CNTD1	0.31	AOAH	0.31	CRYGS	0.3
LOC100270804	0.5	RASGRP1	0.43	GPR162	0.4	WDR27	0.38	IRF1	0.36	RNF166	0.35	PIBF1	0.33	CCDC88A	0.32	QRICH2	0.31	TSPYL4	0.31	BIRC3	0.3
MARCH1	0.5	CFB	0.43	IKZF1	0.4	TRAF3IP3	0.38	SF3B1	0.36	ATP11C	0.35	CCDC76	0.33	KIAA1109	0.32	TOP3B	0.31	KIAA0892	0.31	ARRB2	0.3
LOC162632	0.49	CD72	0.43	NMI	0.4	ATG12	0.38	ADCY7	0.36	ZDHHC17	0.35	PPP1R2	0.33	PUM2	0.32	ZFP37	0.31	YPEL5	0.31	CSF3R	0.3
CCDC112	0.49	STAT4	0.43	STAC3	0.4	MSL3	0.38	BZRAP1	0.36	GIMAP4	0.35	PI4KAP2	0.33	SLC25A27	0.32	FAM135A	0.31	EBI3	0.31	ZNF395	0.3
NSUN6	0.49	PLEKHO1	0.43	HCLS1	0.4	AHSA2	0.38	UBXN4	0.36	MLKL	0.35	RBMS3	0.33	ERO1LB	0.32	HACE1	0.31	CBX7	0.31	EML5	0.3
PLCB1	0.49	NCRNA00120	0.43	BTK	0.39	MITD1	0.38	LOC100133991	0.36	ARHGEF3	0.35	KCTD12	0.33	PDE4B	0.32	RBL2	0.31	STX10	0.31	DOCK4	0.3
CLSTN3	0.48	CYTIP	0.43	ATAD2B	0.39	ICA1	0.38	DOT1L	0.36	TSPAN15	0.35	LAIR1	0.33	HAVCR2	0.32	SFRS2	0.31	AZI2	0.31	HLA-DQA2	0.3
SEMA4D	0.48	PNRC1	0.43	MYO1F	0.39	LOC100128288	0.38	SGEF	0.36	RBM43	0.35	TRAFD1	0.33	RGPD6	0.32	ETAA1	0.31	INPP4A	0.31	IKZF4	0.3

EXCLI Journal 2024;23:1375-1396 – ISSN 1611-2156
Received: August 08, 2024, accepted: November 06, 2024, published: November 19, 2024

C1S	0.48	ZNF621	0.43	CCDC57	0.39	APOL6	0.38	AHI1	0.36	LILRB2	0.35	EVL	0.33	PHKA2	0.32	FAM48A	0.31	RNASE6	0.31	SFRS17A	0.3
RASGRP2	0.48	CXCL13	0.42	TNFRSF19	0.39	CCR2	0.38	ITGA10	0.36	HESX1	0.35	IKZF5	0.33	MAT2B	0.32	DMTF1	0.31	KSR1	0.31	ZNF235	0.3
LRRC48	0.48	ANKZF1	0.42	GPR18	0.39	RAB33B	0.37	THSD1P1	0.36	COL16A1	0.35	GIMAP8	0.33	TET2	0.32	ANKHD1	0.31	TADA1	0.31	MBOAT1	0.3
DDX17	0.48	CCDC130	0.42	L3MBTL	0.39	LOC338799	0.37	SYNE1	0.36	RUFY2	0.35	CALML4	0.33	SCAMP1	0.32	ANKRD13A	0.31	SORCS1	0.31	ZGLP1	0.3
TBX19	0.48	CYTH4	0.42	PHIP	0.39	TBC1D3B	0.37	NUDT13	0.36	PM20D2	0.35	SFRS5	0.33	C16orf45	0.32	ZCCHC11	0.31	USF1	0.31	JHDM1D	0.3
FAM22A	0.48	DOCK2	0.42	ZNF443	0.39	CRLF3	0.37	RBMS1	0.36	C7orf10	0.35	PARVG	0.33	MYO15B	0.32	KLF13	0.31	LAPTM5	0.31	REV1	0.3
NFKBID	0.47	TNFSF13B	0.42	TUBD1	0.39	OGFRL1	0.37	ZAP70	0.36	MIER1	0.34	C9orf72	0.33	HP1BP3	0.32	FAM36A	0.31	CNST	0.31	TTC32	0.3
LCA5	0.47	CTGF	0.42	FAM160B1	0.39	EMB	0.37	LOC200030	0.36	HDAC7	0.34	CEP192	0.33	IL15RA	0.32	CSGAL-NACT2	0.31	UBE2J1	0.3	LRRC6	0.3
FAM22D	0.47	TBC1D8	0.42	HLA-DOB	0.39	TMSL3	0.37	TNFAIP2	0.36	GCC2	0.34	MRC2	0.33	LOC100286793	0.32	PIAS3	0.31	KLRG1	0.3	ING5	0.3
MLLT6	0.47	FGD2	0.42	COL12A1	0.39	CMTM1	0.37	C12orf4	0.36	SERP1	0.34	IBTK	0.33	ANKMY1	0.32	DTX3L	0.31	ARHGDIB	0.3	C12orf75	0.3
TNFAIP8	0.47	CYP27B1	0.42	PAQR6	0.39	C5orf54	0.37	FLJ45340	0.36	RAB5A	0.34	KGFLP2	0.33	CHD2	0.32	RAB11FIP2	0.31	FAM156A	0.3	NRF1	0.3
GPR65	0.47	TMC8	0.42	CLK1	0.39	PIK3AP1	0.37	EFNA5	0.36	WSB1	0.34	KCNMB3	0.33	TCERG1	0.32	LOC100271722	0.31	CROCCL1	0.3	RBBP6	0.3
GOLGA6L10	0.47	AGXT2L2	0.42	LOH3CR2A	0.39	INO80D	0.37	WDFY4	0.36	ANKRD36	0.34	ZNF780B	0.33	NCOA1	0.32	PARP11	0.31	MAPK11	0.3	FKBP5	0.3
THAP2	0.47	CSF2RB	0.42	SMAP1	0.39	DGKD	0.37	PPIEL	0.36	CHURC1	0.34	CD14	0.33	CDC23	0.32	DRAM1	0.31	STAMBPL1	0.3	ZNF75D	0.3
LOC642826	0.47	AMPD3	0.42	AASS	0.39	SLC25A34	0.37	CHML	0.36	TPR	0.34	FEM1C	0.33	LRRC27	0.32	ABI1	0.31	CAV2	0.3	SKIL	0.3
APBB3	0.47	LILRB1	0.42	MRI1	0.39	NISCH	0.37	NFYB	0.36	APOL4	0.34	C6orf134	0.33	ALMS1	0.32	ZNF674	0.31	ERICH1	0.3	FAM13B	0.3
LOC399959	0.47	CPT1B	0.42	SEC31B	0.39	C7orf63	0.37	IL6	0.36	EFCAB7	0.34	SPOPL	0.33	POLG2	0.32	USP34	0.31	UPF2	0.3	CCDC102A	0.3
SENP7	0.47	IL2RA	0.42	PRKAB2	0.39	ARHGAP30	0.37	DENND1B	0.36	MBNL1	0.34	CD2AP	0.33	RBM33	0.32	PRR5L	0.31	SFXN5	0.3	C14orf181	0.3
TNFRSF6B	0.47	DOK3	0.42	STK11IP	0.39	ZNF343	0.37	C6orf26	0.36	AMT	0.34	KIAA2026	0.33	ARHGAP33	0.32	SIGLEC10	0.31	ZBTB46	0.3	ATG16L2	0.3
DMPK	0.46	GCA	0.42	CRBN	0.39	FAM134B	0.37	CEP170	0.36	TRANK1	0.34	ELF2	0.33	LOC388152	0.32	C20orf12	0.31	C1QC	0.3	KIAA1324L	0.3
CD180	0.46	TAGAP	0.42	PPWD1	0.39	SLC2A5	0.37	TICAM2	0.36	GSDMB	0.34	ZNF799	0.33	CASP7	0.32	LOC400027	0.31	DOCK8	0.3	YIPF5	0.3
GIMAP7	0.46	FLI1	0.42	OFD1	0.39	GIMAP6	0.37	DND1	0.36	CLIP4	0.34	SLCO2B1	0.33	AIF1	0.32	ELL2	0.31	MAP3K14	0.3	ZNF37A	0.3
MZF1	0.46	GOLGA6L9	0.42	CHRNB1	0.39	SLAMF6	0.37	AGAP6	0.36	PRPF38B	0.34	ARHGAP15	0.33	EZH1	0.32	ETV6	0.31	ITGAM	0.3	LRRK2	0.3
CD47	0.46	ZBTB40	0.42	TANK	0.39	LOC146880	0.37	ACCN3	0.36	CDK19	0.34	CCDC14	0.33	CLU	0.32	ARL13B	0.31	NCBP2	0.3	C1orf131	0.3
DENND3	0.46	LOC284900	0.42	ITGA2	0.39	PTPN6	0.37	SLC22A4	0.36	NCF4	0.34	GADD45B	0.33	FGD6	0.32	XPC	0.31	C10orf18	0.3	MYST4	0.3
MANEA	0.46	RPGR	0.42	POC5	0.39	GULP1	0.37	ALOX5	0.36	CCDC66	0.34	SNX22	0.33	ARL6IP6	0.32	GCH1	0.31	QRSL1	0.3	RELB	0.3

EXCLI Journal 2024;23:1375-1396 – ISSN 1611-2156
Received: August 08, 2024, accepted: November 06, 2024, published: November 19, 2024

RABL2A	0.46	BRD8	0.42	DCP2	0.39	TRPM8	0.37	ATG5	0.36	KIAA0528	0.34	C5orf41	0.33	TTC30B	0.32	IFT88	0.31	C1orf213	0.3	GRAP	0.3
CHRD	0.46	IP6K2	0.42	FRY	0.39	MS4A6A	0.37	MCM9	0.36	FZD5	0.34	UBLCP1	0.33	MYLK	0.32	CALCOCO1	0.31	FEZ2	0.3	VGLL4	0.3
CACNB1	0.46	ALS2CR8	0.42	NFKBIZ	0.39	SENP6	0.37	XPA	0.36	ZNF763	0.34	METTL10	0.33	PLCB2	0.32	ADD3	0.31	C6orf162	0.3	MARCH8	0.3
NLGN2	0.46	HAPLN3	0.42	PAPD4	0.39	ACAP1	0.37	CSAD	0.36	PRKCI	0.34	SDHAP1	0.33	CEP135	0.32	KIFAP3	0.31	HMGN4	0.3	PBX3	0.3
PTGER4	0.46	C2orf27A	0.42	CLEC7A	0.39	FCGR3A	0.37	CCDC121	0.36	RUNX1	0.34	TTC7A	0.33	GON4L	0.32	CSTF2T	0.31	CD300A	0.3	NCRNA00174	0.3
C5orf53	0.46	SNX20	0.42	CYBB	0.39	C1orf104	0.37	APOL3	0.36	KIAA1274	0.34	UBE2B	0.33	C5orf15	0.32	NCRNA00085	0.31	C17orf65	0.3	APOBEC3H	0.3
CCNL1	0.45	ZNF292	0.42	SIGLEC14	0.39	TXNDC15	0.37	DNHD1	0.35	PRKAR2B	0.34	LOC80154	0.33	TRERF1	0.32	DHFRL1	0.31	KCNAB1	0.3	AP3S1	0.3
ING4	0.45	C9orf45	0.41	MNDA	0.39	GOPC	0.37	HNRNPH1	0.35	RNF44	0.34	PCM1	0.33	EPC1	0.32	CD37	0.31	TRMT11	0.3	JMJD1C	0.3
PCDHB10	0.45	DFFB	0.41	PCDHB2	0.39	ARHGAP9	0.37	SPRN	0.35	ALCAM	0.34	C12orf51	0.33	FAM26F	0.32	WDR11	0.31	BBX	0.3		
ARHGEF6	0.45	WDR5B	0.41	SMC5	0.39	SEMA6C	0.37	SGMS1	0.35	CYP2D7P1	0.34	ZNF638	0.33	LAMB1	0.32	C3orf63	0.31	PPP3CB	0.3		
ZNF44	0.45	TMEM67	0.41	RNASET2	0.39	TTC21A	0.37	PLEKHF2	0.35	C17orf100	0.34	CDKL3	0.33	WBP4	0.32	GPR183	0.31	BAZ2B	0.3		
ADORA2A	0.45	GRAP2	0.41	N4BP2L2	0.39	IFT81	0.37	TXNIP	0.35	PWWP2A	0.34	GIMAP1	0.33	JAK2	0.32	FAM32A	0.31	CDK5RAP3	0.3		
TTC39C	0.45	EFHC1	0.41	SLC11A1	0.39	CD53	0.37	CORO1A	0.35	CCDC69	0.34	SERTAD2	0.33	NCF1	0.32	ARHGAP27	0.31	TFPI	0.3		
RHOH	0.45	LIF	0.41	BTAF1	0.39	HK3	0.37	IQSEC1	0.35	FAM178A	0.34	MST1P2	0.33	PURA	0.32	DPYSL3	0.31	FAM117B	0.3		
LIPT1	0.45	PCDHB6	0.41	LENG8	0.39	ACAD11	0.37	GNRHR2	0.35	ZBTB26	0.34	ATXN7	0.33	MBTD1	0.32	VEZT	0.31	ZNF506	0.3		
TRPV1	0.45	AFTPH	0.41	ZRANB2	0.39	NEGR1	0.37	CP110	0.35	ANUBL1	0.34	SPAST	0.33	CLEC2D	0.32	SMG1	0.31	LMBRD1	0.3		
PTK2B	0.45	KIAA0753	0.41	BCAS4	0.39	LY6G5B	0.37	LOC100129550	0.35	RCAN1	0.34	MAP3K7	0.33	SLTM	0.32	PRICKLE4	0.31	SMAD7	0.3		
CAPRIN2	0.45	KLRA1	0.41	ZNF333	0.39	SMAD5	0.37	PHOSPHO2	0.35	YTHDC2	0.34	CCDC50	0.33	C19orf36	0.32	SLC25A36	0.31	NAPEPLD	0.3		
SOD2	0.45	C20orf96	0.41	ZFC3H1	0.39	AGGF1	0.37	PGGT1B	0.35	DGCR8	0.34	C4A	0.33	ZNF20	0.32	LUC7L3	0.31	VPS13A	0.3		
RFXAP	0.45	CERKL	0.41	XBP1	0.39	GPR132	0.37	CCDC91	0.35	C2orf68	0.34	ETF1	0.33	MBD4	0.32	ERAP1	0.31	FANCF	0.3		
SCHIP1	0.45	PCNXL2	0.41	FAM113B	0.39	SCAND2	0.37	DZIP1L	0.35	WAS	0.34	RBCK1	0.33	ZNF300	0.32	DPY19L2	0.31	FAP	0.3		
ZNF248	0.45	GABBR1	0.41	FYB	0.39	C10orf118	0.37	ANKS3	0.35	PLCG2	0.34	BCL3	0.33	GPR34	0.32	PUS10	0.31	C16orf46	0.3		
C3orf62	0.45	RHOU	0.41	IDI2	0.39	SCARF1	0.37	MATR3	0.35	GIMAP5	0.34	VHL	0.33	TRPS1	0.32	C3orf19	0.31	DDX5	0.3		
PLEK	0.45	EVI2B	0.41	ZNF177	0.39	ZNF652	0.37	IQCB1	0.35	RGPD1	0.34	SLC4A5	0.33	COG3	0.32	OGFOD2	0.31	ZBTB11	0.3		
IRF8	0.45	TUBGCP6	0.41	C4orf7	0.39	CCDC122	0.37	WTAP	0.35	NDFIP1	0.34	KIAA0776	0.33	C2orf64	0.32	LMO4	0.31	APPL2	0.3		

EXCLI Journal 2024;23:1375-1396 – ISSN 1611-2156
Received: August 08, 2024, accepted: November 06, 2024, published: November 19, 2024

**Negatively correlated with FER1L4**																					
**Gene**	**R**	**Gene**	**R**	**Gene**	**R**	**Gene**	**R**	**Gene**	**R**	**Gene**	**R**	**Gene**	**R**	**Gene**	**R**	**Gene**	**R**	**Gene**	**R**	**Gene**	**R**
SLC3A2	-0.33	ATP6V1F	-0.32	SILV	-0.31	CLCN7	-0.31	SCYL1	-0.31	RAB38	-0.31	POLR2L	-0.31	SLC45A2	-0.31	SCARB1	-0.3	TBC1D16	-0.3	TAF10	-0.3
BACE2	-0.33	GYPC	-0.31	C6orf125	-0.31	ATP6V0C	-0.31	G6PC3	-0.31	SLC25A39	-0.31	HMOX2	-0.31								

Supplementary Table 3: Involvement of genes positively and negatively correlated (R-Pearson <-0.3 and >0.3) with *FER1L4* in molecular pathways and processes based on REACTOME pathway analysis. Only genes with p < 0.05 were indicated.

FER1L4			
**Pathway name involved with genes positively correlated**	**Genes number**	**Pathway name involved with genes negatively correlated**	**Genes number**
RUNX3 Regulates Immune Response and Cell Migration	6	Iron uptake and transport	3
Interleukin-10 signaling	18	Insulin receptor recycling	2
Cilium Assembly	31	Transport of small molecules	6
Rho GTPase cycle	25	Transferrin endocytosis and recycling	2
Activation of C3 and C5	4	Severe congenital neutropenia type 4 (G6PC3)	1
Anchoring of the basal body to the plasma membrane	17	ROS and RNS production in phagocytes	2
Organelle biogenesis and maintenance	42	RNA Polymerase II Transcription Initiation And Promoter Clearance	2
RUNX1 regulates estrogen receptor mediated transcription	4	RNA Polymerase II HIV Promoter Escape	2
Interleukin-2 family signaling	10	RNA Polymerase II Promoter Escape	2
Intraflagellar transport	10	RNA Polymerase II Transcription Pre-Initiation and Promoter Opening	2
Interleukin-3, Interleukin-5 and GMCSF signaling	9	HIV Transcription Initiation	2
Interleukin receptor SHC signaling	6	RNA Polymerase II Transcription Initiation	2
Loss of proteins required for interphase microtubule organization from the centrosome	11	Defective SLC7A7 causes lysinuric protein intolerance (LPI)	1
Loss of Nlp from mitotic centrosomes	11	Ion channel transport	3
Nef and signal transduction	3	Amino acids regulate mTORC1	2
Recruitment of mitotic centrosome proteins and complexes	12	Transcription of the HIV genome	2
RUNX3 regulates RUNX1-mediated transcription	2	HDL clearance	1
Regulation of TP53 Expression	2	Melanin biosynthesis	1
Interleukin-35 Signalling	4	RNA Polymerase II Pre-transcription Events	2
CD28 co-stimulation	7	Signaling by Insulin receptor	2

EXCLI Journal 2024;23:1375-1396 – ISSN 1611-2156
Received: August 08, 2024, accepted: November 06, 2024, published: November 19, 2024

Centrosome maturation	12	Scavenging by Class B Receptors	1
AURKA Activation by TPX2	11	Beta-oxidation of pristanoyl-CoA	1
TRIF-mediated programmed cell death	3	Rab regulation of trafficking	2
Signal regulatory protein family interactions	4	Synthesis of bile acids and bile saltsvia 24-hydroxycholesterol	1
YAP1- and WWTR1 (TAZ)-stimulated gene expression	4	Tryptophan catabolism	1

Supplementary Table 4: Involvement of gene sets significantly ($p < 0.05$) enriched in group of patients with high and low *FER1L4* expression phenotypes with regard to hallmarks (H) and oncogenic (C6) gene sets based on GSEA analysis; SIZE - number of enriched genes in specified process/pathway; NES - normalized enrichment score; FDR q-value - false discovery rate q-value

Gene	Data set	Group	FDR qvalue	p value	Process
FER1L4	Hallmarks	low	0.126	0.013	MYC Targets
			0.156	0.035	Oxidative phosphorylation
		high	0.199	0.036	Inflammatory response
			0.149	0.032	IL6 JAK STAT3 signaling
			0.210	0.034	IL2 STAT5 signaling
	Oncogenic signatures	high	0.006	0.216	KRAS.LUNG_UP.V1_UP
			0.002	0.222	JAK2_DN.V1_DN
			0.008	0.199	ATF2_UP.V1_DN
			0.012	0.215	STK33_SKM_UP
			0.014	0.264	MTOR_UP.N4.V1_DN
			0.012	0.226	KRAS.600.LUNG.BREAST_UP.V1_UP
			0.016	0.229	KRAS.LUNG.BREAST_UP.V1_UP
			0.010	0.214	SRC_UP.V1_DN
			0.012	0.205	CSR_EARLY_UP.V1_DN
			0.010	0.197	PKCA_DN.V1_DN
			0.030	0.198	STK33_UP
			0.065	0.200	RPS14_DN.V1_UP
			0.029	0.214	MYC_UP.V1_DN
			0.026	0.209	PRC1_BMI_UP.V1_UP
			0.046	0.224	PTEN_DN.V2_UP
			0.021	0.216	KRAS.600_UP.V1_UP

EXCLI Journal 2024;23:1375-1396 – ISSN 1611-2156
Received: August 08, 2024, accepted: November 06, 2024, published: November 19, 2024

Supplementary Table 5: The list of genes enriched in the pathways IL2/STAT5 SIGNALING, IL6/JAK/STAT3 SIGNALING and INFLAMMATORY RESPONSE in patients with high expression level of *FER1L4*, which were used as gene sets for disease free survival and overall survival analysis using GEPIA2 database.

FER1L4		
**IL2/STAT5 SIGNALING**	**IL6/JAK/STAT3 SIGNALING**	**INFLAMMATORY RESPONSE**
CSF1	GRB2	IL10
SPRY4	CRLF2	INHBA
TGM2	IL17RB	GNA15
CST7	IL1R2	PDPN
ITGAE	PIM1	PDE4B
IL3RA	IL13RA1	CHST2
GADD45B	STAT3	IL4R
TLR7	IL4R	BDKRB1
NFIL3	CSF2RA	TNFSF15
HOPX	ITGB3	FPR1
GLIPR2	IL10RB	ITGB3
ABCB1	IL9R	ABCA1
ADAM19	PLA2G2A	C5AR1
GPR83	TNF	IFITM1
CCR4	IFNAR1	HPN
ICOS	CCR1	IFNAR1
PHTF2	CSF3R	MXD1
SH3BGRL2	IL1R1	CMKLR1
RORA	STAT1	ADM
TTC39B	CSF1	MEP1A
BMP2	IL3RA	CLEC5A
NRP1	ACVRL1	CSF3R
SNX14	IL12RB1	IL1R1
ITIH5	PIK3R5	LYN
ITGAV	SOCS3	C3AR1
LIF	IL1B	CSF1
IL2RB	CD14	TIMP1
FLT3LG	DNTT	TNFAIP6
TNFRSF1B	STAM2	MARCO
NFKBIZ	IL6	MSR1
SPP1	EBI3	MEFV
RGS16	TNFRSF1B	EDN1
NDRG1	CCL7	LCP2
CDC42SE2	IFNGR1	GABBR1
IFNGR1	LTB	CCRL2

EXCLI Journal 2024;23:1375-1396 – ISSN 1611-2156
Received: August 08, 2024, accepted: November 06, 2024, published: November 19, 2024

LTB	IL2RG	AXL
BATF3	SOCS1	CCL5
NCOA3	CXCL9	PIK3R5
FAM126B	CXCL10	ACVR2A
IKZF4	IRF1	SERPINE1
TRAF1	IL7	ICAM4
LRIG1	CXCL11	VIP
TNFRSF9	PTPN2	CCR7
SOCS1	OSMR	LDLR
IL10RA	CSF2RB	OSM
SCN9A	IL15RA	ICOSLG
IRF8	CXCL13	IL1B
TNFRSF8	IL2RA	CD14
CXCL10	IL18R1	NFKBIA
CD86	FAS	CALCRL
SELL	LEPR	STAB1
CD79B	MAP3K8	LIF
AHR	PDGFC	IL6
COCH	ITGA4	IL2RB
TNFRSF18	CD38	CXCR6
XBP1	TLR2	EBI3
IL2RA		NLRP3
IL18R1		TNFRSF1B
TNFRSF4		CCL7
IKZF2		GP1BA
PTGER2		CYBB
CASP3		LCK
CCND2		IL12B
TNFSF10		RGS16
DHRS3		ATP2B1
TNFSF11		NDP
GPR65		TLR1
MAP3K8		PTGIR
GBP4		CD70
RHOH		SLC31A2
CD48		GCH1
FGL2		TNFRSF9
AGER		IL10RA
PLAGL1		F3
		CXCL9
		CXCL10
		IRF1

EXCLI Journal 2024;23:1375-1396 – ISSN 1611-2156
Received: August 08, 2024, accepted: November 06, 2024, published: November 19, 2024

		CCL2
		IL7R
		SELL
		IL18
		LTA
		LAMP3
		CXCL11
		PTGER4
		AHR
		OSMR
		PTPRE
		NOD2
		CD40
		GPR132
		NMUR1
		IL15RA
		IL18R1
		PTGER2
		TNFSF10
		GPR183
		SCARF1
		IRAK2
		SEMA4D
		IL18RAP
		BTG2
		SLAMF1
		LPAR1
		CD48
		KCNA3
		RASGRP1
		SLC1A2
		IL15
		TLR2
		NMI
		CD69

EXCLI Journal 2024;23:1375-1396 – ISSN 1611-2156
Received: August 08, 2024, accepted: November 06, 2024, published: November 19, 2024

Supplementary Table 6: Involvement of gene sets presented in Supplementary Table 5 in specified processes/pathways based on GeneMANIA online tool; FDR - false discovery rate

FER1L4			
**IL2/STAT5 SIGNALING**			
**Function**	**FDR**	**Genes in network**	**Genes in genome**
regulation of leukocyte differentiation	7.502378693647688e-9	14	192
cytokine receptor activity	4.608430773953404e-8	10	80
positive regulation of cell adhesion	6.041314635118648e-8	15	299
cell surface	6.041314635118648e-8	14	251
regulation of T cell activation	6.041314635118648e-8	14	252
receptor complex	9.696270893074563e-8	13	217
immune receptor activity	9.696270893074563e-8	11	132
positive regulation of lymphocyte activation	1.040573220479204e-7	13	221
regulation of hemopoiesis	1.5758177371049998e-7	14	283
positive regulation of hemopoiesis	1.609874140238337e-7	10	107
positive regulation of leukocyte differentiation	1.609874140238337e-7	10	107
cellular response to tumor necrosis factor	4.1735809997201447e-7	11	159
positive regulation of leukocyte activation	4.1735809997201447e-7	13	257
regulation of lymphocyte differentiation	4.6801570225104035e-7	10	122
positive regulation of cell activation	4.805339338972153e-7	13	263
positive regulation of cell-cell adhesion	5.926350746234549e-7	12	216
response to tumor necrosis factor	0.0000015345639736342548	12	236
lymphocyte differentiation	0.000001595267085636852	12	238
leukocyte cell-cell adhesion	0.000007399557669014962	12	274
regulation of leukocyte proliferation	0.00001300233500028516	10	178
regulation of T cell differentiation	0.000017486039746618616	8	96
positive regulation of leukocyte cell-cell adhesion	0.000018927881025043885	10	187
cytokine binding	0.000022059783726872998	8	100
regulation of leukocyte cell-cell adhesion	0.000029050648905105662	11	254
T cell differentiation	0.00003078716677328479	9	149
plasma membrane signaling receptor complex	0.00005779836285409106	9	161
positive regulation of T cell activation	0.00005870259166932057	9	162
regulation of mononuclear cell proliferation	0.00006627154242419446	9	165
leukocyte migration	0.00006714409165520592	11	281
leukocyte proliferation	0.00006714409165520592	10	221
myeloid leukocyte migration	0.0001448829962588663	9	183
receptor signaling pathway via STAT	0.00019501641829190557	8	138
mononuclear cell proliferation	0.0003134080882625861	9	202

positive regulation of cell migration	0.00043065027005717	10	274
positive regulation of lymphocyte differentiation	0.0005791766714477905	6	70
regulation of lymphocyte proliferation	0.0006154359197277134	8	163
regulation of leukocyte migration	0.000656466350985833	8	165
granulocyte migration	0.001053356792605483	7	123
adaptive immune response	0.0015265742742188156	8	186
regulation of B cell activation	0.00159613529599655	6	85
positive regulation of stress-activated MAPK cascade	0.0017842930251100488	6	87
lymphocyte proliferation	0.0022520471734045417	8	198
growth factor binding	0.003017598352315743	6	96
positive regulation of stress-activated protein kinase signaling cascade	0.003322959056983431	6	98
regulatory T cell differentiation	0.0033570945141098364	4	27
dendritic cell differentiation	0.0033570945141098364	4	27
regulation of ERK1 and ERK2 cascade	0.00380999205801661	8	216
cell chemotaxis	0.004264168149283217	8	220
granulocyte chemotaxis	0.004441594863377635	6	105
positive regulation of leukocyte migration	0.00450620465505752	6	106
positive regulation of chemotaxis	0.00450620465505752	6	106
positive regulation of leukocyte proliferation	0.005186495905284657	6	109
I-kappaB kinase/NF-kappaB signaling	0.005331343053168515	8	230
B cell activation	0.005571943048930271	7	167
regulation of tyrosine phosphorylation of STAT protein	0.005647594463195878	5	66
immunoglobulin secretion	0.0059380844930655985	3	11
tyrosine phosphorylation of STAT protein	0.006172784150984635	5	68
ERK1 and ERK2 cascade	0.006172784150984635	8	238
adaptive immune response based on somatic recombination of immune receptors built from immunoglobulin superfamily domains	0.006172784150984635	7	172
receptor signaling pathway via JAK-STAT	0.006412519195300972	6	116
response to interleukin-2	0.007126504350898071	3	12
cellular response to interleukin-2	0.007126504350898071	3	12
positive regulation of leukocyte chemotaxis	0.008071584845817428	5	73
regulation of alpha-beta T cell activation	0.010295717901765247	5	77
leukocyte chemotaxis	0.010616523213104738	7	190
response to lipopolysaccharide	0.013725442457929742	6	135
cellular response to molecule of bacterial origin	0.015456262127001108	6	139
response to interferon-gamma	0.015456262127001108	6	139
myeloid leukocyte differentiation	0.015456262127001108	6	139

EXCLI Journal 2024;23:1375-1396 – ISSN 1611-2156
Received: August 08, 2024, accepted: November 06, 2024, published: November 19, 2024

cellular response to interferon-gamma	0.016955691571754687	5	87
regulation of interleukin-12 production	0.01729159336923159	4	45
mononuclear cell migration	0.017412318296887122	5	88
regulation of mononuclear cell migration	0.018352541163554472	4	46
regulation of stress-activated MAPK cascade	0.019532874310164077	6	147
cytokine receptor binding	0.019532874310164077	7	214
B cell proliferation	0.020595919673433622	4	48
positive regulation of B cell activation	0.020595919673433622	4	48
integral component of plasma membrane	0.02118853757580599	8	295
neuroinflammatory response	0.021241646827094195	4	49
regulation of inflammatory response	0.021241646827094195	7	219
interleukin-12 production	0.021241646827094195	4	49
response to interleukin-7	0.022613302113652967	3	19
cellular response to interleukin-7	0.022613302113652967	3	19
cellular calcium ion homeostasis	0.022613302113652967	8	300
production of molecular mediator of immune response	0.022955735362910318	6	155
stress-activated protein kinase signaling cascade	0.02330364090032035	7	225
regulation of stress-activated protein kinase signaling cascade	0.023793192295572214	6	157
neutrophil migration	0.023793192295572214	5	98
peptidyl-tyrosine phosphorylation	0.023793192295572214	7	227
regulation of JNK cascade	0.02559253166678169	5	100
lymphocyte costimulation	0.02575318545648607	4	53
cellular response to biotic stimulus	0.02613099605874351	6	161
alpha-beta T cell activation	0.027195471631981556	5	102
positive regulation of immune effector process	0.027364232213954842	6	163
regulation of leukocyte chemotaxis	0.03020129972226272	5	105
regulation of production of molecular mediator of immune response	0.03020129972226272	5	105
positive regulation of amyloid precursor protein catabolic process	0.0308007794620652	3	22
regulation of cytosolic calcium ion concentration	0.035510431541427405	7	246
regulation of adaptive immune response based on somatic recombination of immune receptors built from immunoglobulin superfamily domains	0.03800762658192074	5	111
regulation of chemotaxis	0.039937691913524934	6	177
peptidyl-tyrosine modification	0.039937691913524934	7	252
myeloid cell differentiation	0.052497484590568674	7	264
bone resorption	0.05362902015524702	3	27

EXCLI Journal 2024;23:1375-1396 – ISSN 1611-2156
Received: August 08, 2024, accepted: November 06, 2024, published: November 19, 2024

positive regulation of immunoglobulin production	0.05362902015524702	3	27
regulation of adaptive immune response	0.05562142856530064	5	122
regulation of NIK/NF-kappaB signaling	0.05822095523989506	4	68
regulation of peptidyl-tyrosine phosphorylation	0.0583177079038477	6	192
response to ketone	0.0621998672649105	4	70
positive regulation of alpha-beta T cell differentiation	0.0621998672649105	3	29
negative regulation of cell adhesion	0.0621998672649105	6	196
regulation of bone remodeling	0.0621998672649105	3	29
response to molecule of bacterial origin	0.0621998672649105	6	195
CD4-positive, alpha-beta T cell activation	0.063889181254191	4	71
positive regulation of tumor necrosis factor superfamily cytokine production	0.063889181254191	4	71
chemokine binding	0.06548590346841607	3	30
regulation of p38MAPK cascade	0.06548590346841607	3	30
positive regulation of CD4-positive, alpha-beta T cell activation	0.06548590346841607	3	30
regulation of cytokine-mediated signaling pathway	0.06633108480489322	5	130
regulation of I-kappaB kinase/NF-kappaB signaling	0.07055062655298895	6	203
positive regulation of production of molecular mediator of immune response	0.07482976086162477	4	75
JNK cascade	0.08223996059412084	5	137
leukocyte activation involved in inflammatory response	0.08352667250033323	3	33
stress-activated MAPK cascade	0.08373086345889674	6	211
gliogenesis	0.08573113015021384	5	139
astrocyte differentiation	0.08910330582277726	3	34
regulation of cell morphogenesis	0.09472077391742902	6	217
**IL6/JAK/STAT3 SIGNALING**			
**Function**	**FDR**	**Genes in network**	**Genes in genome**
immune receptor activity	3.502463587072124e-42	30	132
cytokine receptor activity	9.192717599855866e-41	26	80
cytokine receptor binding	1.356925505744705e-23	23	214
cytokine binding	3.4858771695246136e-20	17	100
receptor signaling pathway via STAT	9.043253706959798e-18	17	138
cell surface	3.296920414668623e-16	19	251
response to interferon-gamma	3.3031577362756236e-16	16	139
regulation of response to cytokine stimulus	9.083976888264696e-16	16	149

EXCLI Journal 2024;23:1375-1396 – ISSN 1611-2156
Received: August 08, 2024, accepted: November 06, 2024, published: November 19, 2024

regulation of cytokine-mediated signaling pathway	3.775812338088533e-15	15	130
receptor complex	3.1977724583802177e-13	16	217
regulation of tyrosine phosphorylation of STAT protein	2.1487985030094392e-12	11	66
plasma membrane signaling receptor complex	2.429818699636072e-12	14	161
tyrosine phosphorylation of STAT protein	2.579467239492046e-12	11	68
regulation of leukocyte proliferation	8.54811320662049e-12	14	178
interferon-gamma-mediated signaling pathway	1.932804064527577e-11	8	23
regulation of response to interferon-gamma	1.932804064527577e-11	8	23
response to molecule of bacterial origin	2.3981953427290305e-11	14	195
receptor signaling pathway via JAK-STAT	2.3981953427290305e-11	12	116
positive regulation of cell adhesion	2.6238970981400568e-11	16	299
cellular response to interferon-gamma	2.891194212846196e-11	11	87
regulation of T cell activation	3.514411472229921e-11	15	252
positive regulation of cell-cell adhesion	8.019197008374533e-11	14	216
leukocyte cell-cell adhesion	1.0475062406976273e-10	15	274
leukocyte proliferation	1.0475062406976273e-10	14	221
cellular response to molecule of bacterial origin	1.56075188461116e-10	12	139
growth factor receptor binding	2.0838728112956938e-10	11	106
positive regulation of leukocyte proliferation	2.744656693077499e-10	11	109
response to interleukin-7	3.157307593902356e-10	7	19
cellular response to interleukin-7	3.157307593902356e-10	7	19
regulation of leukocyte cell-cell adhesion	5.423602448082879e-10	14	254
regulation of receptor signaling pathway via STAT	6.033407193208859e-10	10	85
positive regulation of leukocyte activation	6.033407193208859e-10	14	257
cellular response to interleukin-6	6.363739184278402e-10	7	21
cellular response to biotic stimulus	6.705343505519707e-10	12	161
positive regulation of cell activation	7.463917268833183e-10	14	263
regulation of mononuclear cell proliferation	8.487549934253873e-10	12	165
positive regulation of lymphocyte activation	1.3621138404809546e-9	13	221
leukocyte migration	1.68463356985305e-9	14	281
growth factor binding	1.7255390206110857e-9	10	96
peptidyl-tyrosine phosphorylation	1.7700908205522824e-9	13	227
response to lipopolysaccharide	1.947876672150964e-9	11	135
response to interleukin-15	2.072353832511386e-9	6	13
cellular response to interleukin-15	2.072353832511386e-9	6	13
adaptive immune response	2.863184528188901e-9	12	186
lymphocyte differentiation	2.863184528188901e-9	13	238

positive regulation of leukocyte cell-cell adhesion	2.9319286523216853e-9	12	187
positive regulation of leukocyte migration	3.839921975061528e-9	10	106
regulation of peptidyl-tyrosine phosphorylation	3.839921975061528e-9	12	192
T cell differentiation	4.814335813795969e-9	11	149
peptidyl-tyrosine modification	5.189937224677725e-9	13	252
neuroinflammatory response	5.189937224677725e-9	8	49
mononuclear cell proliferation	6.4298613877371285e-9	12	202
response to interleukin-6	8.689582405249784e-9	7	31
positive regulation of T cell activation	1.0870765359641826e-8	11	162
regulation of lymphocyte proliferation	1.1410749971750575e-8	11	163
regulation of leukocyte migration	1.279270421978787e-8	11	165
regulation of inflammatory response	1.508578147154028e-8	12	219
regulation of hemopoiesis	1.946002387085132e-8	13	283
integral component of plasma membrane	3.2029869855133e-8	13	295
myeloid leukocyte migration	3.6533705820591e-8	11	183
positive regulation of lymphocyte proliferation	4.477014142406773e-8	9	98
chemokine production	5.1192798396069075e-8	8	66
leukocyte chemotaxis	5.206872866390803e-8	11	190
positive regulation of mononuclear cell proliferation	5.477500157039677e-8	9	101
regulation of leukocyte differentiation	5.477500157039677e-8	11	192
positive regulation of T cell cytokine production	5.477500157039677e-8	5	10
positive regulation of peptidyl-tyrosine phosphorylation	5.477500157039677e-8	9	101
regulation of leukocyte chemotaxis	7.29163186414064e-8	9	105
granulocyte chemotaxis	7.29163186414064e-8	9	105
lymphocyte proliferation	7.29163186414064e-8	11	198
regulation of receptor signaling pathway via JAK-STAT	1.2772718493174982e-7	8	75
response to interleukin-2	1.521853652109011e-7	5	12
cellular response to interleukin-2	1.521853652109011e-7	5	12
regulation of interferon-gamma production	1.521853652109011e-7	8	77
positive regulation of cell migration	1.521853652109011e-7	12	274
cytokine production involved in immune response	1.822626765650463e-7	8	79
cell chemotaxis	2.0317554194055446e-7	11	220
interferon-gamma production	2.4038754332931575e-7	8	82
granulocyte migration	2.6771692367559033e-7	9	123
positive regulation of inflammatory response	2.746058983646348e-7	7	52
adaptive immune response based on somatic recombination of immune receptors	2.792391942415467e-7	10	172

EXCLI Journal 2024;23:1375-1396 – ISSN 1611-2156
Received: August 08, 2024, accepted: November 06, 2024, published: November 19, 2024

built from immunoglobulin superfamily domains			
regulation of chemotaxis	3.6456849034985946e-7	10	177
mononuclear cell migration	3.9995817911517785e-7	8	88
ERK1 and ERK2 cascade	4.2700791752602104e-7	11	238
CD4-positive, alpha-beta T cell cytokine production	5.045769160821327e-7	5	15
T cell cytokine production	6.29211636084593e-7	6	33
gliogenesis	7.206516235232004e-7	9	139
regulation of T cell differentiation	7.593063482049897e-7	8	96
regulation of chemokine production	7.830020038753891e-7	7	61
regulation of T cell mediated immunity	0.0000010804364468457028	7	64
cellular response to chemokine	0.0000010804364468457028	7	64
alpha-beta T cell activation	0.000001179179737461363	8	102
regulation of cytokine production involved in immune response	0.0000013172525837662795	7	66
regulation of leukocyte mediated immunity	0.000001383787411037766	9	151
regulation of phagocytosis	0.000001435552293037894	7	67
positive regulation of chemotaxis	0.000001535065690881564	8	106
positive regulation of hemopoiesis	0.0000016202611187422918	8	107
positive regulation of leukocyte differentiation	0.0000016202611187422918	8	107
regulation of T-helper 1 type immune response	0.000001657439140287536	5	19
response to interleukin-1	0.0000020000740217743895	9	159
positive regulation of tumor necrosis factor superfamily cytokine production	0.0000020000740217743895	7	71
regulation of ERK1 and ERK2 cascade	0.0000020000740217743895	10	216
response to chemokine	0.0000020000740217743895	7	71
positive regulation of leukocyte chemotaxis	0.000002412027355679597	7	73
regulation of lymphocyte mediated immunity	0.000002677503359974572	8	115
T cell mediated immunity	0.000002865188587708777	7	75
positive regulation of T cell mediated immunity	0.0000031168962611482706	6	44
cytokine activity	0.000003552273101879716	6	45
regulation of lymphocyte differentiation	0.000004072004881053658	8	122
regulation of adaptive immune response	0.000004072004881053658	8	122
T-helper 1 type immune response	0.000004224463590240754	5	23
response to tumor necrosis factor	0.000004224463590240754	10	236
T cell proliferation	0.0000061410883712021925	8	129
regulation of neuroinflammatory response	0.00000645906448165627	5	25
response to macrophage colony-stimulating factor	0.0000072253213493082805	4	10
tissue remodeling	0.000007429272473715992	7	87
regulation of T cell cytokine production	0.00000776850314214872	5	26

positive regulation of interleukin-1 production	0.000007954873719926707	6	52
regulation of innate immune response	0.000008255901902783823	10	255
myeloid cell differentiation	0.000011336045838497848	10	264
phagocytosis	0.000012222083257382656	9	200
CD4-positive, alpha-beta T cell differentiation	0.000012222083257382656	6	56
neutrophil migration	0.00001603753460905937	7	98
chemokine receptor binding	0.000018166987115562164	6	60
glial cell differentiation	0.000019439838947182186	7	101
temperature homeostasis	0.000020751173254820167	8	153
regulation of acute inflammatory response	0.000021518036233853696	5	32
production of molecular mediator of immune response	0.00002257945985692771	8	155
T cell activation involved in immune response	0.000023484485405433127	6	63
regulation of production of molecular mediator of immune response	0.00002443409161808693	7	105
leukocyte activation involved in inflammatory response	0.000024512412446971916	5	33
positive regulation of defense response	0.000029888888083036413	10	296
regulation of tumor necrosis factor production	0.000030884509249502625	7	109
angiogenesis	0.00003133374514399985	10	298
lymphocyte chemotaxis	0.00003234012097759782	5	35
regulation of adaptive immune response based on somatic recombination of immune receptors built from immunoglobulin superfamily domains	0.00003421632912404424	7	111
foam cell differentiation	0.00003636609569259108	5	36
glial cell activation	0.00003636609569259108	5	36
positive regulation of cytokine production involved in immune response	0.00003636609569259108	5	36
positive regulation of myeloid cell differentiation	0.00003670046603644409	6	69
positive regulation of adaptive immune response based on somatic recombination of immune receptors built from immunoglobulin superfamily domains	0.00003670046603644409	6	69
alpha-beta T cell differentiation	0.00003670046603644409	6	69
positive regulation of adaptive immune response	0.00003670046603644409	6	69
phosphatidylinositol 3-kinase regulator activity	0.000036961642482550405	4	15
homeostasis of number of cells	0.000038523796980909704	7	114
regulation of interleukin-8 production	0.00003919670998697955	6	70
CD4-positive, alpha-beta T cell activation	0.00004239424908153006	6	71
regulation of interleukin-1 beta production	0.000045796045204819454	6	72
tumor necrosis factor production	0.000047450259450444766	7	118

EXCLI Journal 2024;23:1375-1396 – ISSN 1611-2156
Received: August 08, 2024, accepted: November 06, 2024, published: November 19, 2024

regulation of T cell proliferation	0.0000495957399410808	7	119
regulation of tumor necrosis factor superfamily cytokine production	0.0000495957399410808	7	119
lymphocyte mediated immunity	0.0000503647203749383 7	8	176
interleukin-8 production	0.0000522062994004629 2	6	74
positive regulation of lymphocyte mediated immunity	0.0000601255898739040 7	6	76
interleukin-1 beta production	0.0000601255898739040 7	6	76
tumor necrosis factor superfamily cytokine production	0.0000601255898739040 7	7	123
regulation of alpha-beta T cell activation	0.0000645216836445877 5	6	77
astrocyte development	0.0000945422535407661 2	4	19
regulation of interleukin-1 production	0.0000995879571907436 8	6	83
bone remodeling	0.0000996563330857308 4	5	45
regulation of mononuclear cell migration	0.0001100980674968876 9	5	46
cellular response to interleukin-12	0.0001100980674968876 9	5	46
CD4-positive, alpha-beta T cell differentiation involved in immune response	0.0001220819852590169 6	5	47
myeloid leukocyte differentiation	0.0001308036123376891 3	7	139
regulation of tissue remodeling	0.0001309429811394599	5	48
response to type I interferon	0.0001309429811394599	6	88
cellular response to type I interferon	0.0001309429811394599	6	88
positive regulation of alpha-beta T cell activation	0.0001309429811394599	5	48
response to interleukin-12	0.0001309429811394599	5	48
alpha-beta T cell differentiation involved in immune response	0.0001309429811394599	5	48
alpha-beta T cell activation involved in immune response	0.0001309429811394599	5	48
positive regulation of leukocyte mediated immunity	0.0001391679288982008 6	6	89
regulation of lymphocyte chemotaxis	0.0001606031511894139 5	4	22
regulation of membrane protein ectodomain proteolysis	0.0001606031511894139 5	4	22
interleukin-1 production	0.0001663170986808655 6	6	92
T cell differentiation involved in immune response	0.0001710790272174036	5	51
acute inflammatory response	0.0001876453758329237 4	5	52
positive T cell selection	0.0001884319863435749 8	4	23
membrane protein ectodomain proteolysis	0.0001884319863435749 8	4	23
regulation of interleukin-6 production	0.0002076747873193132 4	6	96
positive regulation of gliogenesis	0.0002229669761473528	4	24
cellular response to interleukin-1	0.0002460020093782715 4	6	99
regulation of vasculature development	0.0002659784038971488	8	225

EXCLI Journal 2024;23:1375-1396 – ISSN 1611-2156
Received: August 08, 2024, accepted: November 06, 2024, published: November 19, 2024

cellular response to tumor necrosis factor	0.00028653758952519554	7	159
interleukin-6 production	0.0002881843403237736	6	102
negative regulation of cell junction assembly	0.00030348574716972115	4	26
glial cell development	0.00030754913812156795	5	58
positive regulation of immune effector process	0.0003307452342521534	7	163
lipid storage	0.0003314737920946459	5	59
T cell selection	0.00034592689885975164	4	27
regulatory T cell differentiation	0.00034592689885975164	4	27
muscle cell proliferation	0.0003651482671437896	6	107
regulation of myeloid cell differentiation	0.0003651482671437896	7	166
positive regulation of DNA-binding transcription factor activity	0.0004233709832957425	7	170
macrophage activation	0.0004456801247272515	5	63
positive regulation of alpha-beta T cell differentiation	0.0004535527238717588	4	29
chemokine binding	0.0005165322119218775	4	30
positive regulation of CD4-positive, alpha-beta T cell activation	0.0005165322119218775	4	30
positive regulation of lymphocyte migration	0.0005824993169481789	4	31
regulation of macrophage derived foam cell differentiation	0.0005824993169481789	4	31
macrophage derived foam cell differentiation	0.0005824993169481789	4	31
cellular response to leptin stimulus	0.0006299937361608367	3	10
negative regulation of receptor signaling pathway via JAK-STAT	0.0006299937361608367	3	10
maintenance of location	0.0007125902175179065	8	261
positive regulation of lymphocyte differentiation	0.0007148143597334056	5	70
regulation of reactive oxygen species metabolic process	0.0007661634486927594	6	123
regulation of smooth muscle cell proliferation	0.0008095630609081037	5	72
positive regulation of protein secretion	0.0008095630609081037	5	72
astrocyte differentiation	0.0008184727364368971	4	34
positive regulation of ion transport	0.000840373477581937	7	191
regulation of osteoclast differentiation	0.000912653172594998	4	35
positive regulation of production of molecular mediator of immune response	0.0009710254088713747	5	75
cellular response to peptide	0.0009724169597733286	8	274
positive regulation of T cell proliferation	0.0010093887193178728	4	36
negative regulation of gene silencing by miRNA	0.0010744823429484653	3	12
regulation of glial cell proliferation	0.0010744823429484653	3	12
response to leptin	0.0010744823429484653	3	12

EXCLI Journal 2024;23:1375-1396 – ISSN 1611-2156
Received: August 08, 2024, accepted: November 06, 2024, published: November 19, 2024

regulation of myeloid leukocyte differentiation	0.001074530716654406	5	77
positive regulation of myeloid leukocyte differentiation	0.0011026748517505107	4	37
smooth muscle cell proliferation	0.001134284986606034	5	78
positive regulation of response to cytokine stimulus	0.0012067660607752303	4	38
negative regulation of lipid localization	0.0012067660607752303	4	38
regulation of CD4-positive, alpha-beta T cell differentiation	0.0012067660607752303	4	38
regulation of gliogenesis	0.0013229635122064586	4	39
cytokine secretion	0.0013229635122064586	4	39
positive regulation of receptor signaling pathway via STAT	0.0013229635122064586	4	39
T-helper cell lineage commitment	0.001331586655489476	3	13
lymphocyte activation involved in immune response	0.0013372197732734424	6	138
regulation of lipid storage	0.0014401835911152978	4	40
nitric oxide biosynthetic process	0.0014401835911152978	4	40
regulation of phosphatidylinositol 3-kinase signaling	0.0015448961329648193	5	84
myeloid cell homeostasis	0.0015448961329648193	5	84
regulation of cold-induced thermogenesis	0.0016018197409283967	6	143
regulation of nitric-oxide synthase biosynthetic process	0.001618733652374243	3	14
positive regulation of proteolysis	0.001618733652374243	8	298
regulation of amyloid-beta clearance	0.001618733652374243	3	14
nitric-oxide synthase biosynthetic process	0.001618733652374243	3	14
cold-induced thermogenesis	0.0016313801376012114	6	144
cellular response to lipopolysaccharide	0.001847491551592218	4	43
T-helper cell differentiation	0.001847491551592218	4	43
adaptive thermogenesis	0.0018814023529659543	6	148
lymphocyte migration	0.002303338121182664	5	92
negative regulation of gene silencing by RNA	0.00238595368870799	3	16
glial cell proliferation	0.00238595368870799	3	16
negative regulation of posttranscriptional gene silencing	0.00238595368870799	3	16
CD4-positive, alpha-beta T cell lineage commitment	0.00238595368870799	3	16
regulation of alpha-beta T cell differentiation	0.0027893774573008344	4	48
CD4-positive or CD8-positive, alpha-beta T cell lineage commitment	0.002820402523838267	3	17
alpha-beta T cell lineage commitment	0.002820402523838267	3	17
macrophage differentiation	0.002820402523838267	3	17
negative regulation of receptor signaling pathway via STAT	0.002820402523838267	3	17

inositol lipid-mediated signaling	0.002820402523838267	6	160
regulation of lymphocyte migration	0.002956651549586374	4	49
osteoclast differentiation	0.0031917736774072255	4	50
regulation of regulatory T cell differentiation	0.003309432816612861	3	18
T cell lineage commitment	0.003309432816612861	3	18
positive regulation of lymphocyte chemotaxis	0.003309432816612861	3	18
B cell activation	0.003464566616320843	6	167
positive regulation of T cell differentiation	0.0036441239338953174	4	52
regulation of CD4-positive, alpha-beta T cell activation	0.0036441239338953174	4	52
endothelium development	0.0036785083105622873	5	103
phosphatidylinositol 3-kinase signaling	0.0038380315937979284	5	104
positive regulation of apoptotic process	0.003870576355802027	6	171
membrane protein proteolysis	0.003870576355802027	4	53
nitric oxide metabolic process	0.004448167191878376	4	55
negative regulation of extrinsic apoptotic signaling pathway	0.004448167191878376	4	55
cellular extravasation	0.0047584967337204135	4	56
reactive nitrogen species metabolic process	0.00505430882088521	4	57
regulation of production of small RNA involved in gene silencing by RNA	0.00505430882088521	3	21
positive regulation of CD4-positive, alpha-beta T cell differentiation	0.00505430882088521	3	21
T-helper 17 cell differentiation	0.00505430882088521	3	21
regulation of production of miRNAs involved in gene silencing by miRNA	0.00505430882088521	3	21
positive regulation of amyloid precursor protein catabolic process	0.0058147578829938805	3	22
cell-substrate adhesion	0.006030962101431176	7	271
regulation of reactive oxygen species biosynthetic process	0.006062749865270367	4	60
reactive oxygen species metabolic process	0.006622982471953014	6	190
regulation of lipid localization	0.00717120241293914	5	120
T-helper 17 type immune response	0.007436514944232549	3	24
regulation of heterotypic cell-cell adhesion	0.007436514944232549	3	24
regulation of epithelial cell apoptotic process	0.007668625583798012	4	64
negative regulation of cell adhesion	0.007716615990415789	6	196
positive regulation of peptide secretion	0.007886996296675186	5	123
humoral immune response	0.007886996296675186	6	197
G protein-coupled receptor binding	0.008071865312780103	6	198
positive regulation of monooxygenase activity	0.008220912312433562	3	25
negative regulation of cellular response to insulin stimulus	0.008220912312433562	3	25

EXCLI Journal 2024;23:1375-1396 – ISSN 1611-2156
Received: August 08, 2024, accepted: November 06, 2024, published: November 19, 2024

amyloid-beta clearance	0.00913963401999603	3	26
regulation of angiogenesis	0.00913963401999603	6	203
regulation of vascular endothelial growth factor production	0.00913963401999603	3	26
CCR chemokine receptor binding	0.00913963401999603	3	26
phosphatidylinositol-mediated signaling	0.009252241138706383	5	128
regulation of NIK/NF-kappaB signaling	0.009316026353707572	4	68
positive regulation of secretion by cell	0.00945953859979728	6	205
bone resorption	0.010011941394975429	3	27
regulation of alpha-beta T cell proliferation	0.010011941394975429	3	27
vascular endothelial growth factor production	0.010011941394975429	3	27
positive regulation of nitric oxide metabolic process	0.010011941394975429	3	27
membrane microdomain	0.010066748483915599	6	208
epithelial cell apoptotic process	0.0107602208716225	4	71
cellular response to external stimulus	0.010980596140614947	6	212
interleukin-17 production	0.010980596140614947	3	28
alpha-beta T cell proliferation	0.010980596140614947	3	28
regulation of interleukin-17 production	0.010980596140614947	3	28
erythrocyte homeostasis	0.01117320425894672	4	72
positive regulation of secretion	0.011772422316164894	6	215
regulation of bone remodeling	0.012032903384835914	3	29
negative regulation of gene silencing	0.012032903384835914	3	29
positive regulation of reactive oxygen species biosynthetic process	0.01324658037157715	3	30
regulation of amyloid-beta formation	0.01324658037157715	3	30
positive regulation of nervous system development	0.013650335854772244	5	141
cellular response to peptide hormone stimulus	0.014065818245909557	6	223
amyloid-beta formation	0.014438355350456712	3	31
negative regulation of T cell differentiation	0.014438355350456712	3	31
regulation of protein secretion	0.014618069173002363	6	225
regulation of cell killing	0.015430480510905462	4	79
positive regulation of endopeptidase activity	0.015692872011105155	5	146
heterotypic cell-cell adhesion	0.015692872011105155	3	32
endothelial cell differentiation	0.016826980307190435	4	81
regulation of cytokine secretion	0.0169939574018321	3	33
regulation of cytokine production involved in inflammatory response	0.0169939574018321	3	33
positive regulation of type I interferon production	0.0169939574018321	3	33
negative regulation of nervous system development	0.018248229993767302	4	83

programmed necrotic cell death	0.018414861476719644	3	34
response to corticosteroid	0.018414861476719644	3	34
reactive oxygen species biosynthetic process	0.019744936058009865	4	85
regulation of B cell activation	0.019744936058009865	4	85
negative regulation of peptidyl-tyrosine phosphorylation	0.019840588767106107	3	35
negative regulation of endothelial cell migration	0.019840588767106107	3	35
positive regulation of cysteine-type endopeptidase activity involved in apoptotic process	0.020459685564104168	4	86
regulation of cell-substrate adhesion	0.02046813619268482	5	156
response to peptide hormone	0.020486071757123307	6	242
positive regulation of calcium ion transport into cytosol	0.0210855097621321	3	36
regulation of insulin receptor signaling pathway	0.0210855097621321	3	36
regulation of nervous system development	0.0210855097621321	6	244
regulation of amyloid precursor protein catabolic process	0.0210855097621321	3	36
regulation of nitric oxide biosynthetic process	0.0210855097621321	3	36
NIK/NF-kappaB signaling	0.021806385228441288	4	88
positive regulation of peptidase activity	0.021811367194213643	5	159
regulation of cytosolic calcium ion concentration	0.021826384538234985	6	246
phosphatidylinositol metabolic process	0.02231578520996053	5	160
negative regulation of lymphocyte differentiation	0.02246507843350211	3	37
amyloid-beta metabolic process	0.02246507843350211	3	37
regulation of cellular response to insulin stimulus	0.02425229761139684	3	38
regulation of calcium ion transport	0.025392626388393562	5	165
regulation of peptide secretion	0.025413619553851234	6	254
epithelial cell migration	0.025872225625340972	6	255
leukocyte adhesion to vascular endothelial cell	0.02589865641103781	3	39
regulation of gene silencing by miRNA	0.027681824270736177	3	40
cytokine production involved in inflammatory response	0.027681824270736177	3	40
necrotic cell death	0.027681824270736177	3	40
epithelium migration	0.028833808700656247	6	261
response to mechanical stimulus	0.02905751674989338	4	96
regulation of B cell proliferation	0.02944812905231439	3	41
production of miRNAs involved in gene silencing by miRNA	0.02944812905231439	3	41
antimicrobial humoral response	0.02996399790960513	4	97

EXCLI Journal 2024;23:1375-1396 – ISSN 1611-2156
Received: August 08, 2024, accepted: November 06, 2024, published: November 19, 2024

protein secretion	0.030106510679594165	6	264
positive regulation of protein transport	0.030106510679594165	6	264
positive regulation of oxidoreductase activity	0.031098632536228157	3	42
tissue migration	0.031098632536228157	6	266
regulation of granulocyte chemotaxis	0.031098632536228157	3	42
positive regulation of cell development	0.031630066920997	5	175
amyloid precursor protein catabolic process	0.03315446393256572	3	43
positive regulation of type 2 immune response	0.03515343796793867	2	10
endothelial cell apoptotic process	0.03519046795763617	3	44
negative regulation of cytokine-mediated signaling pathway	0.03519046795763617	3	44
positive regulation of establishment of protein localization	0.03568461723545184	6	274
positive regulation of neurogenesis	0.03614909873952684	4	103
positive regulation of small molecule metabolic process	0.03614909873952684	4	103
regulation of epithelial cell differentiation	0.03614909873952684	4	103
regulation of neurogenesis	0.03685319059587558	5	182
negative regulation of ERBB signaling pathway	0.03689239747053246	3	45
regulation of interleukin-12 production	0.03689239747053246	3	45
positive regulation of cation transmembrane transport	0.03844096811801734	4	105
signal transduction in absence of ligand	0.0390424263237466	3	46
insulin receptor signaling pathway	0.0390424263237466	3	46
regulation of type I interferon production	0.03951483666287629	4	106
fat-soluble vitamin biosynthetic process	0.0411572487447312	2	11
vitamin D metabolic process	0.0411572487447312	2	11
calcium-mediated signaling	0.04204598475026517	4	108
positive regulation of B cell activation	0.043405223575753576	3	48
B cell proliferation	0.043405223575753576	3	48
regulation of metal ion transport	0.043405223575753576	6	287
negative regulation of cell development	0.043405223575753576	4	109
T cell migration	0.045617865248133375	3	49
regulation of extrinsic apoptotic signaling pathway	0.045617865248133375	4	111
interleukin-12 production	0.045617865248133375	3	49
negative regulation of response to cytokine stimulus	0.045617865248133375	3	49
hemostasis	0.04685255853281666	6	292
coagulation	0.04685724991145269	6	293

EXCLI Journal 2024;23:1375-1396 – ISSN 1611-2156
Received: August 08, 2024, accepted: November 06, 2024, published: November 19, 2024

regulation of cysteine-type endopeptidase activity involved in apoptotic signaling pathway	0.04685724991145269	2	12
positive regulation of acute inflammatory response	0.04685724991145269	2	12
regulation of monooxygenase activity	0.04685724991145269	3	50
positive regulation of steroid biosynthetic process	0.04685724991145269	2	12
regulation of interleukin-5 production	0.04685724991145269	2	12
phosphatidylinositol 3-kinase complex	0.04685724991145269	2	12
regulation of MHC class II biosynthetic process	0.04685724991145269	2	12
negative regulation of protein phosphorylation	0.04685724991145269	6	293
MHC class II biosynthetic process	0.04685724991145269	2	12
positive regulation of reactive oxygen species metabolic process	0.04685724991145269	3	50
kinase regulator activity	0.04754959977238733	5	196
neuron death	0.04826618159850586	5	197
response to virus	0.04826618159850586	5	197
cellular response to insulin stimulus	0.04826618159850586	4	114
epithelial cell development	0.04826618159850586	4	114
production of small RNA involved in gene silencing by RNA	0.048792423266589995	3	51
dsRNA processing	0.048792423266589995	3	51
type I interferon production	0.04949313842897743	4	115
negative regulation of epithelial cell migration	0.051133062386718435	3	52
cellular calcium ion homeostasis	0.051133062386718435	6	300
positive regulation of calcium ion transmembrane transport	0.051133062386718435	3	52
positive regulation of ion transmembrane transport	0.05225475100945054	4	117
interleukin-5 production	0.053284488333664476	2	13
regulation of epithelial cell migration	0.053848683627154226	5	203
endothelial cell proliferation	0.058618079062171737	4	121
cell-matrix adhesion	0.058618079062171737	4	121
amyloid precursor protein metabolic process	0.05918878622765388	3	55
endothelial cell development	0.05918878622765388	3	55
granulocyte macrophage colony-stimulating factor production	0.06082889859936457	2	14
regulation of macrophage differentiation	0.06082889859936457	2	14
regulation of granulocyte macrophage colony-stimulating factor production	0.06082889859936457	2	14
production of molecular mediator involved in inflammatory response	0.061627692931630375	3	56

EXCLI Journal 2024;23:1375-1396 – ISSN 1611-2156
Received: August 08, 2024, accepted: November 06, 2024, published: November 19, 2024

regulation of release of sequestered calcium ion into cytosol	0.061627692931630375	3	56
positive regulation of protein serine/threonine kinase activity	0.0637185282434021	5	212
positive regulation of lipid biosynthetic process	0.06441417167999153	3	57
tumor necrosis factor-mediated signaling pathway	0.06441417167999153	3	57
glycosaminoglycan binding	0.06615611568086727	4	126
insulin secretion	0.06615611568086727	4	126
positive regulation of cysteine-type endopeptidase activity	0.06795076507555499	4	127
T cell chemotaxis	0.06838383232780731	2	15
morphogenesis of an endothelium	0.06838383232780731	2	15
regulation of toll-like receptor signaling pathway	0.07018183688352804	3	59
negative regulation of cell-cell adhesion	0.07335349009273646	4	130
regulation of cell junction assembly	0.07527076272649268	4	131
vitamin biosynthetic process	0.07723944654627175	2	16
carbohydrate homeostasis	0.07920186770121372	4	133
positive regulation of cellular amide metabolic process	0.08121609461035817	4	134
regulation of endothelial cell development	0.08632515622347288	2	17
response to interferon-beta	0.08632515622347288	2	17
positive regulation of T-helper cell differentiation	0.08632515622347288	2	17
I-kappaB kinase/NF-kappaB signaling	0.0875946499932751	5	230
cell killing	0.09178163895320261	4	139
positive regulation of protein catabolic process	0.09399493159897919	4	140
growth hormone receptor signaling pathway	0.09556746292365852	2	18
regulation of CD8-positive, alpha-beta T cell activation	0.09556746292365852	2	18
positive regulation of gene silencing by miRNA	0.09556746292365852	2	18
release of sequestered calcium ion into cytosol	0.09776001195953998	3	67
**INFLAMMATORY RESPONSE**			
**Function**	**FDR**	**Genes in network**	**Genes in genome**
cellular response to molecule of bacterial origin	1.0579295862963435e-24	25	139
response to molecule of bacterial origin	3.686293208163624e-24	27	195
immune receptor activity	3.686293208163624e-24	24	132
cellular response to biotic stimulus	1.2924939675328212e-23	25	161

EXCLI Journal 2024;23:1375-1396 – ISSN 1611-2156
Received: August 08, 2024, accepted: November 06, 2024, published: November 19, 2024

response to lipopolysaccharide	1.5078270152490333e-22	23	135
leukocyte migration	1.6736492062116553e-21	28	281
leukocyte chemotaxis	9.178012165669254e-18	22	190
cell chemotaxis	1.001234544001024e-17	23	220
myeloid leukocyte migration	7.347830156723003e-17	21	183
cytokine receptor binding	8.951906360967302e-17	22	214
regulation of inflammatory response	1.3518630492321409e-16	22	219
cell surface	2.417348787146285e-15	22	251
positive regulation of cell activation	6.108259612009389e-15	22	263
cytokine receptor activity	7.02336160698682e-15	15	80
granulocyte migration	8.237110463384699e-15	17	123
regulation of leukocyte proliferation	1.1477197519475056e-14	19	178
granulocyte chemotaxis	1.4210505713220764e-14	16	105
regulation of cytosolic calcium ion concentra-tion	1.7830148359842073e-14	21	246
positive regulation of lymphocyte activation	3.1690289354028386e-14	20	221
leukocyte proliferation	3.1690289354028386e-14	20	221
positive regulation of leukocyte activation	3.749273723394303e-14	21	257
regulation of mononuclear cell proliferation	4.1328369941134167e-14	18	165
receptor signaling pathway via STAT	4.1328369941134167e-14	17	138
mononuclear cell proliferation	8.378506492235943e-14	19	202
leukocyte cell-cell adhesion	1.1625248502943422e-13	21	274
positive regulation of cell-cell adhesion	2.711578650068317e-13	19	216
regulation of lymphocyte proliferation	5.899587254031988e-13	17	163
positive regulation of cell adhesion	6.061399181259412e-13	21	299
cellular calcium ion homeostasis	6.25866377069853e-13	21	300
lymphocyte proliferation	8.292170703605807e-13	18	198
neutrophil migration	2.3353516431494306e-12	14	98
cytokine binding	3.0297069758501703e-12	14	100
regulation of leukocyte cell-cell adhesion	4.268905888896145e-12	19	254
positive regulation of leukocyte cell-cell ad-hesion	4.748464739587131e-12	17	187
regulation of leukocyte migration	1.0735468865524055e-11	16	165
mononuclear cell migration	1.2862791883292765e-11	13	88
response to tumor necrosis factor	1.464912171834251e-11	18	236
ERK1 and ERK2 cascade	1.6514905563509633e-11	18	238
regulation of chemotaxis	2.919792795794151e-11	16	177
blood coagulation	4.044327796058776e-11	16	181
regulation of T cell activation	4.114327375836314e-11	18	252
regulation of leukocyte mediated immunity	4.174521389703097e-11	15	151
regulation of ERK1 and ERK2 cascade	4.174521389703097e-11	17	216
positive regulation of defense response	5.139544257843413e-11	19	296

regulation of leukocyte chemotaxis	1.0872844526459821e-10	13	105
interferon-gamma production	1.1713770633625412e-10	12	82
positive regulation of leukocyte migration	1.1798660586253808e-10	13	106
phagocytosis	1.5999925247343925e-10	16	200
positive regulation of leukocyte proliferation	1.6349386385650593e-10	13	109
cellular response to chemokine	1.7321186236955746e-10	11	64
G protein-coupled receptor signaling path-way, coupled to cyclic nucleotide second messenger	1.7571019206456523e-10	16	202
receptor complex	5.205291383163381e-10	16	217
response to chemokine	5.408662148987631e-10	11	71
regulation of smooth muscle cell proliferation	6.230467018948976e-10	11	72
negative regulation of cytokine production	6.514118270733546e-10	16	221
adaptive immune response	6.902996409996061e-10	15	186
response to interleukin-1	1.160220087897694e-9	14	159
regulation of interferon-gamma production	1.2467136621254494e-9	11	77
smooth muscle cell proliferation	1.4187582613907082e-9	11	78
positive regulation of T cell activation	1.423842701631652e-9	14	162
cytokine production involved in immune re-sponse	1.5852386394950622e-9	11	79
positive regulation of chemotaxis	2.0160351960088177e-9	12	106
chemokine receptor binding	2.2885471654693055e-9	10	60
cellular response to lipopolysaccharide	2.954269360793399e-9	9	43
adaptive immune response based on so-matic recombination of immune receptors built from immunoglobulin superfamily do-mains	2.9747227835874933e-9	14	172
hemostasis	3.5003182148571556e-9	17	292
coagulation	3.5959078926664785e-9	17	293
adenylate cyclase-modulating G protein-cou-pled receptor signaling pathway	3.5959078926664785e-9	14	175
G protein-coupled receptor activity	3.824115956529462e-9	16	252
cytokine activity	4.196938260727685e-9	9	45
positive regulation of T cell cytokine produc-tion	4.596968629027647e-9	6	10
receptor signaling pathway via JAK-STAT	5.05897701083326e-9	12	116
positive regulation of leukocyte mediated im-munity	5.05897701083326e-9	11	89
regulation of tyrosine phosphorylation of STAT protein	5.270583497247233e-9	10	66
interleukin-1 production	7.032000167720495e-9	11	92
tyrosine phosphorylation of STAT protein	7.032000167720495e-9	10	68
neuroinflammatory response	8.648388506069824e-9	9	49
peptidyl-tyrosine phosphorylation	8.697080555924516e-9	15	227

EXCLI Journal 2024;23:1375-1396 – ISSN 1611-2156
Received: August 08, 2024, accepted: November 06, 2024, published: November 19, 2024

regulation of interleukin-8 production	9.075653414340369e-9	10	70
positive regulation of cell migration	1.1579535895719381e-8	16	274
positive regulation of lymphocyte proliferation	1.329466157883e-8	11	98
positive regulation of leukocyte chemotaxis	1.3464345320421245e-8	10	73
positive regulation of inflammatory response	1.4126977145698686e-8	9	52
plasma membrane signaling receptor complex	1.4506825558261711e-8	13	161
interleukin-8 production	1.4934112577457488e-8	10	74
G protein-coupled receptor binding	1.499492360627438e-8	14	198
T cell proliferation	1.4996898591737597e-8	12	129
positive regulation of immune effector process	1.6176839964547626e-8	13	163
positive regulation of mononuclear cell proliferation	1.6687764618103488e-8	11	101
regulation of hemopoiesis	1.6687764618103488e-8	16	283
G protein-coupled peptide receptor activity	1.8395165751548835e-8	11	102
membrane microdomain	2.7028176824451064e-8	14	208
positive regulation of apoptotic process	2.7927881250655564e-8	13	171
muscle cell proliferation	3.010934844445785e-8	11	107
peptidyl-tyrosine modification	3.119982541416501e-8	15	252
response to interferon-gamma	3.2717530715473283e-8	12	139
regulation of tumor necrosis factor production	3.572737315243762e-8	11	109
lymphocyte mediated immunity	3.799012201216084e-8	13	176
regulation of interleukin-1 production	4.1261357321178554e-8	10	83
regulation of adaptive immune response based on somatic recombination of immune receptors built from immunoglobulin superfamily domains	4.226145249572451e-8	11	111
maintenance of location	4.793756388698061e-8	15	261
regulation of B cell activation	5.094234985854881e-8	10	85
myeloid cell differentiation	5.512530213184928e-8	15	264
regulation of lymphocyte mediated immunity	5.973106851318615e-8	11	115
CD4-positive, alpha-beta T cell cytokine production	7.207611553232025e-8	6	15
tumor necrosis factor production	7.74838519561816e-8	11	118
regulation of T cell proliferation	8.332244620794194e-8	11	119
regulation of tumor necrosis factor superfamily cytokine production	8.332244620794194e-8	11	119
regulation of cytokine production involved in immune response	1.0001796928450104e-7	9	66
production of molecular mediator of immune response	1.0154797064081415e-7	12	155
regulation of interleukin-12 production	1.0177824350141978e-7	8	45
regulation of adaptive immune response	1.0514496046277418e-7	11	122

EXCLI Journal 2024;23:1375-1396 – ISSN 1611-2156
Received: August 08, 2024, accepted: November 06, 2024, published: November 19, 2024

tumor necrosis factor superfamily cytokine production	1.1380480318366032e-7	11	123
regulation of mononuclear cell migration	1.192922789009482e-7	8	46
positive regulation of myeloid cell differentiation	1.4271495274515585e-7	9	69
regulation of interleukin-6 production	1.5174825540157826e-7	10	96
interleukin-12 production	1.9753930147216246e-7	8	49
B cell activation	2.2163733573709936e-7	12	167
integral component of plasma membrane	2.2163733573709936e-7	15	295
peptide receptor activity	2.2873902798145635e-7	11	132
positive regulation of peptidyl-tyrosine phosphorylation	2.408871850697771e-7	10	101
regulation of acute inflammatory response	2.439712169370948e-7	7	32
interleukin-6 production	2.6124867775823113e-7	10	102
T cell cytokine production	3.0297138891162813e-7	7	33
acute inflammatory response	3.0338810158841474e-7	8	52
positive regulation of lymphocyte mediated immunity	3.12309539538966e-7	9	76
interleukin-1 beta production	3.12309539538966e-7	9	76
regulation of production of molecular mediator of immune response	3.3428599023549826e-7	10	105
myeloid leukocyte differentiation	3.6915784517676743e-7	11	139
positive regulation of hemopoiesis	3.9336819244550247e-7	10	107
positive regulation of leukocyte differentiation	3.9336819244550247e-7	10	107
protein secretion	4.2608327690898974e-7	14	264
lymphocyte chemotaxis	4.3970938967907824e-7	7	35
cyclic-nucleotide-mediated signaling	4.933573513650703e-7	12	181
foam cell differentiation	5.347770232628526e-7	7	36
negative regulation of response to external stimulus	6.06747219434123e-7	14	272
cAMP-mediated signaling	7.783400067513057e-7	11	150
regulation of leukocyte differentiation	9.272286561787999e-7	12	192
regulation of peptidyl-tyrosine phosphorylation	9.272286561787999e-7	12	192
cytokine secretion	9.344369932489021e-7	7	39
lymphocyte differentiation	0.0000010537569913079957	13	238
NIK/NF-kappaB signaling	0.0000010537569913079957	9	88
T cell activation involved in immune response	0.0000012832284178193366	8	63
cellular response to tumor necrosis factor	0.0000013653493067022617	11	159
regulation of T cell mediated immunity	0.00000142838526580021	8	64
regulation of epithelial cell apoptotic process	0.00000142838526580021	8	64
lymphocyte migration	0.0000015035756217301135	9	92
peptide binding	0.0000015762402278081945	13	247

EXCLI Journal 2024;23:1375-1396 – ISSN 1611-2156
Received: August 08, 2024, accepted: November 06, 2024, published: November 19, 2024

regulation of neuroinflammatory response	0.0000017011937400733326	6	25
regulation of phagocytosis	0.000002014574866958108	8	67
regulation of myeloid cell differentiation	0.0000020412134865177972	11	166
endothelial cell apoptotic process	0.00000207186901204542	7	44
positive regulation of T cell mediated immunity	0.00000207186901204542	7	44
regulation of T cell cytokine production	0.0000021143026004676036	6	26
G protein-coupled chemoattractant receptor activity	0.0000021143026004676036	6	26
positive regulation of adaptive immune response based on somatic recombination of immune receptors built from immunoglobulin superfamily domains	0.0000024399734821614073	8	69
positive regulation of adaptive immune response	0.0000024399734821614073	8	69
positive regulation of DNA-binding transcription factor activity	0.000002498458181043608	11	170
positive regulation of tumor necrosis factor superfamily cytokine production	0.000003012467594658311	8	71
epithelial cell apoptotic process	0.000003012467594658311	8	71
interleukin-17 production	0.000003274123555032571	6	28
regulation of interleukin-17 production	0.000003274123555032571	6	28
regulation of interleukin-1 beta production	0.0000033080656668059835	8	72
lymphocyte activation involved in immune response	0.0000037110107608096654	10	138
gliogenesis	0.000003952078350120817	10	139
positive regulation of production of molecular mediator of immune response	0.0000044753803614023675	8	75
T cell mediated immunity	0.0000044753803614023675	8	75
chemokine binding	0.000004921676817939475	6	30
regulation of macrophage derived foam cell differentiation	0.000005998069072804279	6	31
macrophage derived foam cell differentiation	0.000005998069072804279	6	31
regulation of response to cytokine stimulus	0.000007385599726228878	10	149
leukocyte activation involved in inflammatory response	0.000008820474000636276	6	33
regulation of receptor signaling pathway via STAT	0.000011651961941382583	8	85
angiogenesis	0.000012124791886215536	13	298
toll-like receptor signaling pathway	0.000012124791886215536	9	119
lipid storage	0.00001474428277107227	7	59
glial cell activation	0.00001474428277107227	6	36
positive regulation of cytokine production involved in immune response	0.00001474428277107227	6	36
regulation of T-helper 1 type immune response	0.000014845668750463368	5	19

positive regulation of secretion by cell	0.0000149425637884092 26	11	205
positive regulation of myeloid leukocyte differentiation	0.000017211649785138136	6	37
eosinophil migration	0.000019363513507999625	5	20
positive regulation of secretion	0.000023630314704498344	11	215
positive regulation of receptor signaling pathway via STAT	0.000023630314704498344	6	39
regulation of lipid storage	0.000027349409713581948	6	40
regulation of endopeptidase activity	0.0000295514302980447	12	270
negative regulation of secretion by cell	0.000030164160924991128	8	97
chemokine production	0.000030164160924991128	7	66
leukocyte apoptotic process	0.000030164160924991128	7	66
regulation of lymphocyte chemotaxis	0.00003084297263286634	5	22
dendritic cell migration	0.00003084297263286634	5	22
regulation of membrane protein ectodomain proteolysis	0.00003084297263286634	5	22
regulation of vasculature development	0.00003533646183715227	11	225
regulation of protein secretion	0.00003533646183715227	11	225
calcium ion transport	0.00003566475960521452	12	276
regulation of NIK/NF-kappaB signaling	0.000035807682018097953	7	68
regulation of GTPase activity	0.00003782026175866791	11	227
T-helper 1 type immune response	0.00003782026175866791	5	23
membrane protein ectodomain proteolysis	0.00003782026175866791	5	23
regulation of endothelial cell apoptotic process	0.00003952518347293635	6	43
alpha-beta T cell activation	0.00004155046075048848	8	102
I-kappaB kinase/NF-kappaB signaling	0.00004224540529485497	11	230
chemokine receptor activity	0.000046580516115735434	5	24
positive regulation of endopeptidase activity	0.000059444799657596733	9	146
pattern recognition receptor signaling pathway	0.0000594610398476626	10	190
CD4-positive, alpha-beta T cell differentiation involved in immune response	0.00006580184509352461	6	47
positive regulation of cytosolic calcium ion concentration	0.00006580184509352461	9	148
regulation of receptor signaling pathway via JAK-STAT	0.00006644946018583218	7	75
negative regulation of secretion	0.00006656747618199489	8	109
T cell differentiation	0.00006853925638667275	9	149
monocyte chemotaxis	0.00006861402943693583	5	26
regulation of tissue remodeling	0.00007200808876285182	6	48
B cell proliferation	0.00007200808876285182	6	48
alpha-beta T cell differentiation involved in immune response	0.00007200808876285182	6	48

alpha-beta T cell activation involved in immune response	0.0000720080887628518 2	6	48
positive regulation of proteolysis	0.00007266893477006835	12	298
regulation of peptidase activity	0.00007493346195172406	12	299
regulation of myeloid leukocyte differentiation	0.00007596862697851406	7	77
regulation of MAP kinase activity	0.00008881484121316669	11	250
homeostasis of number of cells	0.00008908124429393013	8	114
T cell differentiation involved in immune response	0.00010067661913326149	6	51
regulation of angiogenesis	0.00010067661913326149	10	203
regulation of peptide secretion	0.00010190248214073486	11	254
positive regulation of peptidase activity	0.00011072236821748924	9	159
positive regulation of interleukin-1 production	0.00011167724838672525	6	52
regulation of phosphatidylinositol 3-kinase signaling	0.00013276051595349843	7	84
regulation of leukocyte apoptotic process	0.00013889694075707625	6	54
positive regulation of protein serine/threonine kinase activity	0.00014487396698608708	10	212
regulation of calcium ion transport	0.00014739474450207196	9	165
negative regulation of extrinsic apoptotic signaling pathway	0.00015303049343241585	6	55
regulation of nitric-oxide synthase biosynthetic process	0.00015399803306225497	4	14
granulocyte macrophage colony-stimulating factor production	0.00015399803306225497	4	14
regulation of granulocyte macrophage colony-stimulating factor production	0.00015399803306225497	4	14
nitric-oxide synthase biosynthetic process	0.00015399803306225497	4	14
leukocyte homeostasis	0.00015491280473444805	5	31
viral life cycle	0.00015661255436196867	11	267
CD4-positive, alpha-beta T cell differentiation	0.00016542317200911585	6	56
phosphatidylinositol-mediated signaling	0.0001978047481132417	8	128
negative regulation of cell population proliferation	0.0002091592258245117	10	222
regulation of cytokine secretion	0.00020961426233207677	5	33
activation of protein kinase activity	0.0002159401355396147	10	223
regulation of cytokine-mediated signaling pathway	0.00021853895054042656	8	130
regulation of toll-like receptor signaling pathway	0.00022024576312154893	6	59
negative regulation of immune system process	0.00024814529735967905	11	281
regulation of chemokine production	0.00026626603480475747	6	61
cargo receptor activity	0.00027561235777985886	5	35
regulation of osteoclast differentiation	0.00027561235777985886	5	35

EXCLI Journal 2024;23:1375-1396 – ISSN 1611-2156
Received: August 08, 2024, accepted: November 06, 2024, published: November 19, 2024

positive regulation of T cell proliferation	0.0003170685059436071	5	36
macrophage activation	0.0003170998213583158	6	63
macrophage differentiation	0.0003374091028963628	4	17
regulation of phospholipase activity	0.0004105333525135375	6	66
positive regulation of response to cytokine stimulus	0.0004105333525135375	5	38
negative regulation of lipid localization	0.0004105333525135375	5	38
negative regulation of B cell activation	0.00042476559453398424	4	18
positive regulation of ion transport	0.0004401225514907006	9	191
platelet activation	0.00044319707426604757	6	67
regulation of gliogenesis	0.0004598174249454722	5	39
negative regulation of defense response	0.0004621121572560037	8	145
homotypic cell-cell adhesion	0.0004778344024643635	6	68
phosphatidylinositol 3-kinase signaling	0.0004844207202831396	7	104
regulation of cysteine-type endopeptidase activity	0.0005086890849029426	9	195
alpha-beta T cell differentiation	0.0005145264715477339	6	69
regulation of type 2 immune response	0.0005149720354972078	4	19
astrocyte development	0.0005149720354972078	4	19
regulation of B cell proliferation	0.0005742290165557306	5	41
CD4-positive, alpha-beta T cell activation	0.000598876949852802	6	71
regulation of natural killer cell activation	0.0006308198949140285	4	20
positive regulation of mononuclear cell migration	0.0006308198949140285	4	20
positive regulation of oxidoreductase activity	0.0006363335235978328	5	42
regulation of granulocyte chemotaxis	0.0006363335235978328	5	42
positive regulation of protein secretion	0.0006377674817306224	6	72
regulation of I-kappaB kinase/NF-kappaB signaling	0.0006751619134333556	9	203
hormone secretion	0.0006997933404713847	9	204
T-helper cell differentiation	0.0007018306720928025	5	43
superoxide metabolic process	0.0007018306720928025	5	43
regulation of extrinsic apoptotic signaling pathway	0.0007018306720928025	7	111
receptor-mediated endocytosis	0.0008468064641820596	8	159
epithelial cell proliferation	0.0008825726213901551	10	266
inositol lipid-mediated signaling	0.0008825726213901551	8	160
hormone transport	0.0008909082476071944	9	211
endocytic vesicle	0.0008909082476071944	9	211
type 2 immune response	0.0008957731087933873	4	22
entry into host	0.0010457189618800192	6	79
lipoprotein particle binding	0.0010673830942371505	4	23
response to lipoprotein particle	0.0010673830942371505	4	23

EXCLI Journal 2024;23:1375-1396 – ISSN 1611-2156
Received: August 08, 2024, accepted: November 06, 2024, published: November 19, 2024

regulation of cell morphogenesis	0.0010945548776304325	9	217
regulation of lipid localization	0.0011207561407553706	7	120
regulation of response to wounding	0.0011207561407553706	7	120
positive regulation of gliogenesis	0.001243677566051626	4	24
regulation of lymphocyte differentiation	0.001243677566051626	7	122
cellular response to lipoprotein particle stimulus	0.001243677566051626	4	24
regulation of heterotypic cell-cell adhesion	0.001243677566051626	4	24
regulation of cysteine-type endopeptidase activity involved in apoptotic process	0.0012465959815759558	8	169
T cell migration	0.0012574434703742467	5	49
regulation of lymphocyte migration	0.0012574434703742467	5	49
negative regulation of leukocyte activation	0.0012805230257852825	7	123
negative regulation of nervous system development	0.0013237849037028861	6	83
osteoclast differentiation	0.0013710996253654632	5	50
positive regulation of reactive oxygen species metabolic process	0.0013710996253654632	5	50
myeloid cell homeostasis	0.0014036650648705411	6	84
negative regulation of leukocyte apoptotic process	0.0014194280191664227	4	25
regulation of monocyte chemotaxis	0.0014194280191664227	4	25
protein-lipid complex binding	0.0014194280191664227	4	25
secretory granule membrane	0.0014712759465024372	9	227
calcium ion transmembrane transport	0.0014774188079165803	8	174
positive regulation of phospholipase activity	0.0014774188079165803	5	51
positive regulation of cysteine-type endopeptidase activity	0.0015192654676027047	7	127
regulation of metal ion transport	0.0015378017543071997	10	287
positive regulation of cysteine-type endopeptidase activity involved in apoptotic process	0.0015540905392726308	6	86
negative regulation of sequestering of calcium ion	0.0015540905392726308	6	86
CCR chemokine receptor binding	0.0016258906306027966	4	26
tissue remodeling	0.001644200153094832	6	87
negative regulation of DNA-binding transcription factor activity	0.001644200153094832	6	87
regulation of epithelial cell proliferation	0.0016909370175049881	9	232
sequestering of calcium ion	0.0017327485898065932	6	88
regulation of sequestering of calcium ion	0.0017327485898065932	6	88
membrane protein proteolysis	0.0017327485898065932	5	53
regulation of alpha-beta T cell proliferation	0.0018569746943270377	4	27
regulation of hormone secretion	0.001874798548146381	8	181

regulation of pattern recognition receptor signaling pathway	0.0019506282844123936	6	90
regulation of lipase activity	0.0020704726475673433	6	91
positive regulation of type 2 immune response	0.0020723934573238113	3	10
regulation of toll-like receptor 2 signaling pathway	0.0020723934573238113	3	10
toll-like receptor 2 signaling pathway	0.0020723934573238113	3	10
alpha-beta T cell proliferation	0.0021088340571558998	4	28
production of molecular mediator involved in inflammatory response	0.002188730996166253	5	56
cellular extravasation	0.002188730996166253	5	56
negative regulation of cell activation	0.002540667939936721	7	139
reactive oxygen species metabolic process	0.0025680810278914928	8	190
glial cell development	0.002573940623900064	5	58
regulation of protein kinase B signaling	0.0026493288050023103	8	191
response to mechanical stimulus	0.0027091799004727557	6	96
regulation of dendritic cell apoptotic process	0.0027343042863381193	3	11
prostanoid receptor activity	0.0027343042863381193	3	11
positive regulation of natural killer cell activation	0.0027343042863381193	3	11
dendritic cell apoptotic process	0.0027343042863381193	3	11
cytosolic calcium ion transport	0.0028335500528248055	7	142
positive regulation of lymphocyte migration	0.0030679833163921653	4	31
cellular response to interleukin-1	0.003154888109200339	6	99
neuron death	0.003199828593147579	8	197
humoral immune response	0.003199828593147579	8	197
extrinsic apoptotic signaling pathway	0.0033311513529331554	7	146
heterotypic cell-cell adhesion	0.0034272890094009553	4	32
positive regulation of phospholipase C activity	0.0034272890094009553	4	32
glial cell differentiation	0.003458090953127577	6	101
regulation of wound healing	0.003458090953127577	6	101
positive regulation of acute inflammatory response	0.003502776583764567	3	12
regulation of glial cell proliferation	0.003502776583764567	3	12
protein kinase B signaling	0.0036013529389472367	8	201
movement in host environment	0.0036124610740741643	6	102
positive regulation of MAP kinase activity	0.0036918149402052564	7	149
negative regulation of epithelial cell apoptotic process	0.003780927531825716	4	33
regulation of cytokine production involved in inflammatory response	0.003780927531825716	4	33
positive regulation of lipase activity	0.0038664116525925474	5	64

EXCLI Journal 2024;23:1375-1396 – ISSN 1611-2156
Received: August 08, 2024, accepted: November 06, 2024, published: November 19, 2024

calcium ion transmembrane import into cytosol	0.003958713999153562	6	104
negative regulation of lymphocyte activation	0.003958713999153562	6	104
astrocyte differentiation	0.004215128974082219	4	34
temperature homeostasis	0.0042780466947431135	7	153
regulation of type I interferon production	0.004353339701502205	6	106
growth factor receptor binding	0.004353339701502205	6	106
response to interleukin-15	0.004356060606753251	3	13
cellular response to interleukin-15	0.004356060606753251	3	13
regulation of viral process	0.004703641371045655	8	210
negative regulation of cell development	0.005027961139492565	6	109
regulation of macrophage differentiation	0.005456634013026092	3	14
response to prostaglandin	0.005456634013026092	3	14
positive regulation of lymphocyte differentiation	0.005733107071928766	5	70
regulation of endothelial cell proliferation	0.005780234850684382	6	112
ossification	0.005998224268594859	8	218
regulation of neuron death	0.00639308422251093	7	164
regulation of symbiotic process	0.006556559759565702	8	221
type I interferon production	0.006615979454037199	6	115
icosanoid receptor activity	0.006642216482255087	3	15
angiogenesis involved in wound healing	0.006642216482255087	3	15
leukocyte adhesion to vascular endothelial cell	0.00691651343842463	4	39
regulation of extrinsic apoptotic signaling pathway via death domain receptors	0.00691651343842463	4	39
stress-activated protein kinase signaling cascade	0.007300495559568478	8	225
cytokine production involved in inflammatory response	0.007604867250598517	4	40
maintenance of location in cell	0.00780071222103466	7	170
microglial cell activation	0.007985564376745547	3	16
glial cell proliferation	0.007985564376745547	3	16
B cell differentiation	0.008295708921389887	4	41
calcium ion transport into cytosol	0.008472848709684481	6	121
endothelial cell proliferation	0.008472848709684481	6	121
regulation of alpha-beta T cell activation	0.008593983258467336	5	77
positive regulation of lipid metabolic process	0.008816761790301948	6	122
macrophage migration	0.009003747703924656	4	42
positive regulation of peptide secretion	0.00914745253858239	6	123
regulation of reactive oxygen species metabolic process	0.00914745253858239	6	123
positive regulation of T-helper cell differentiation	0.009426638029035874	3	17

EXCLI Journal 2024;23:1375-1396 – ISSN 1611-2156
Received: August 08, 2024, accepted: November 06, 2024, published: November 19, 2024

regulation of cell killing	0.009550067517038654	5	79
regulation of interleukin-10 production	0.009749990811985588	4	43
glycosaminoglycan binding	0.010324114745367048	6	126
regulation of interleukin-2 production	0.010615471851146226	4	44
extracellular matrix disassembly	0.011058157444585435	3	18
positive regulation of lymphocyte chemotaxis	0.011058157444585435	3	18
mature B cell differentiation	0.011058157444585435	3	18
response to alcohol	0.011178071931655442	5	82
cell adhesion mediated by integrin	0.011360334499088265	4	45
regulation of phospholipase C activity	0.011360334499088265	4	45
interleukin-2 production	0.011360334499088265	4	45
interleukin-10 production	0.011360334499088265	4	45
regulation of superoxide metabolic process	0.012840938080949285	3	19
detection of another organism	0.012840938080949285	3	19
regulation of G protein-coupled receptor signaling pathway	0.012995881401054763	5	85
extrinsic apoptotic signaling pathway via death domain receptors	0.013327120905678391	4	47
cellular response to interferon-gamma	0.01432258281239978	5	87
erythrocyte differentiation	0.01432258281239978	4	48
positive regulation of stress-activated MAPK cascade	0.01432258281239978	5	87
positive regulation of alpha-beta T cell activation	0.01432258281239978	4	48
positive regulation of macrophage migration	0.01474123365101847	3	20
regulation of macrophage chemotaxis	0.01474123365101847	3	20
negative regulation of blood vessel morphogenesis	0.014942824193231485	5	88
regulation of oxidoreductase activity	0.014942824193231485	5	88
natural killer cell activation	0.015328348348283708	4	49
regulation of innate immune response	0.01543039566163495	8	255
regulation of apoptotic signaling pathway	0.01543039566163495	8	255
osteoblast differentiation	0.015560131961789018	5	89
negative regulation of vasculature development	0.015560131961789018	5	89
regulation of bone mineralization	0.016378022416428058	4	50
positive regulation of CD4-positive, alpha-beta T cell differentiation	0.016748203429899657	3	21
response to virus	0.01723573335947779	7	197
B cell activation involved in immune response	0.017513492549593324	4	51
regulation of blood coagulation	0.017513492549593324	4	51
interaction with host	0.01882659929182344	6	143
negative regulation of wound healing	0.01882659929182344	4	52

EXCLI Journal 2024;23:1375-1396 – ISSN 1611-2156
Received: August 08, 2024, accepted: November 06, 2024, published: November 19, 2024

glial cell migration	0.018894006868557908	3	22
positive regulation of epithelial cell apoptotic process	0.018894006868557908	3	22
positive regulation of leukocyte apoptotic process	0.018894006868557908	3	22
positive regulation of protein transport	0.018894006868557908	8	264
receptor metabolic process	0.019311954142326768	6	144
lymphocyte costimulation	0.01993467465744229	4	53
negative regulation of toll-like receptor signaling pathway	0.02127564249307985	3	23
regulation of leukocyte adhesion to vascular endothelial cell	0.02127564249307985	3	23
detection of external biotic stimulus	0.02127564249307985	3	23
phospholipase C activity	0.02127564249307985	3	23
macrophage chemotaxis	0.02127564249307985	3	23
regulation of hemostasis	0.022613029685545863	4	55
nitric oxide metabolic process	0.022613029685545863	4	55
positive regulation of stress-activated protein kinase signaling cascade	0.023107414161221054	5	98
positive regulation of establishment of protein localization	0.023509381964040805	8	274
negative regulation of inflammatory response	0.023922589628230337	3	24
stress-activated MAPK cascade	0.02475686638771491	7	211
regulation of JNK cascade	0.025117606423008372	5	100
reactive nitrogen species metabolic process	0.025457470583210545	4	57
positive regulation of lipid biosynthetic process	0.025457470583210545	4	57
regulation of coagulation	0.025457470583210545	4	57
positive regulation of monooxygenase activity	0.026571147000470828	3	25
regulation of homotypic cell-cell adhesion	0.026571147000470828	3	25
negative regulation of interleukin-1 production	0.026571147000470828	3	25
negative regulation of peptide secretion	0.026961188335541064	4	58
negative regulation of viral process	0.027006981652077183	5	102
positive regulation of neurogenesis	0.028048009076875175	5	103
regulation of epithelial cell differentiation	0.028048009076875175	5	103
endothelium development	0.028048009076875175	5	103
negative regulation of leukocyte proliferation	0.028457651043343055	4	59
regulation of stress-activated protein kinase signaling cascade	0.028758889199413003	6	157
negative regulation of cell junction assembly	0.029368280232583095	3	26
negative regulation of apoptotic signaling pathway	0.03059697215835377	6	159
regulation of humoral immune response	0.03187567711599034	4	61

EXCLI Journal 2024;23:1375-1396 – ISSN 1611-2156
Received: August 08, 2024, accepted: November 06, 2024, published: November 19, 2024

bone mineralization	0.03187567711599034	4	61
regulation of blood vessel diameter	0.03187567711599034	4	61
bone resorption	0.0323137159774103	3	27
regulation of extrinsic apoptotic signaling pathway in absence of ligand	0.0323137159774103	3	27
positive regulation of nitric oxide metabolic process	0.0323137159774103	3	27
tertiary granule	0.0323137159774103	6	161
cytoplasmic side of plasma membrane	0.032486759158914566	5	107
calcium-mediated signaling	0.03381063232543918	5	108
regulation of T-helper cell differentiation	0.035708361944066784	3	28
extrinsic apoptotic signaling pathway in absence of ligand	0.035708361944066784	3	28
regulation of bone remodeling	0.03929992105340488	3	29
regulation of platelet activation	0.03929992105340488	3	29
positive regulation of alpha-beta T cell differentiation	0.03929992105340488	3	29
positive regulation of cytokine-mediated signaling pathway	0.03929992105340488	3	29
response to fatty acid	0.04309013336332871	3	30
positive regulation of CD4-positive, alpha-beta T cell activation	0.04309013336332871	3	30
detection of biotic stimulus	0.04309013336332871	3	30
positive regulation of reactive oxygen species biosynthetic process	0.04309013336332871	3	30
positive regulation of vasculature development	0.04372986576853445	5	115
release of sequestered calcium ion into cytosol	0.04372986576853445	4	67
regulation of activated T cell proliferation	0.047080517272205226	3	31
regulation of cell adhesion mediated by integrin	0.047080517272205226	3	31
positive regulation of cell development	0.04781032833913768	6	175
response to reactive oxygen species	0.04865829675185341	5	118
regulation of tube size	0.050722503144891656	4	70
regulation of tube diameter	0.050722503144891656	4	70
response to ketone	0.050722503144891656	4	70
regulation of macrophage migration	0.050853830834341834	3	32
regulation of extracellular matrix organization	0.050853830834341834	3	32
regulation of lymphocyte apoptotic process	0.050853830834341834	3	32
regulation of nervous system development	0.05222816973427713	7	244
negative regulation of response to wounding	0.05271697601830517	4	71
positive regulation of apoptotic signaling pathway	0.05271697601830517	4	71
positive regulation of calcium ion transport	0.05271697601830517	4	71

EXCLI Journal 2024;23:1375-1396 – ISSN 1611-2156
Received: August 08, 2024, accepted: November 06, 2024, published: November 19, 2024

regulation of erythrocyte differentiation	0.05499344370540485	3	33
positive regulation of type I interferon production	0.05499344370540485	3	33
erythrocyte homeostasis	0.055196219159584996	4	72
response to oxidative stress	0.056527750193520465	7	248
regulation of neurogenesis	0.05656603214485211	6	182
regulation of osteoblast differentiation	0.057632426117843014	4	73
phosphoric diester hydrolase activity	0.057632426117843014	4	73
regulation of glial cell differentiation	0.0590690624337226	3	34
response to corticosteroid	0.0590690624337226	3	34
activated T cell proliferation	0.0590690624337226	3	34
cellular response to reactive oxygen species	0.06001411060662444	4	74
positive regulation of lipid localization	0.06001411060662444	4	74
mesenchymal cell differentiation	0.06050421813505286	6	185
regulation of ossification	0.06282161855364853	4	75
regulation of epithelial to mesenchymal transition	0.06558424187022978	4	76
regulation of biomineral tissue development	0.06558424187022978	4	76
regulation of biomineralization	0.06558424187022978	4	76
negative regulation of locomotion	0.0658920431703747	7	256
negative regulation of catabolic process	0.06723494367672701	7	257
positive regulation of calcium ion transport into cytosol	0.06804092863475035	3	36
regulation of myeloid leukocyte mediated immunity	0.06804092863475035	3	36
regulation of nitric oxide biosynthetic process	0.06804092863475035	3	36
external side of plasma membrane	0.06804092863475035	3	36
metanephric nephron tubule development	0.0718063492663983	2	10
cellular response to prostaglandin stimulus	0.0718063492663983	2	10
negative regulation of chemokine production	0.0718063492663983	2	10
renal vesicle morphogenesis	0.0718063492663983	2	10
response to macrophage colony-stimulating factor	0.0718063492663983	2	10
nephron tubule epithelial cell differentiation	0.0718063492663983	2	10
dendritic cell antigen processing and presentation	0.0718063492663983	2	10
epithelial cell proliferation involved in liver morphogenesis	0.0718063492663983	2	10
podosome assembly	0.0718063492663983	2	10
vascular associated smooth muscle contraction	0.0718063492663983	2	10
NK T cell activation	0.0718063492663983	2	10
liver morphogenesis	0.0718063492663983	2	10
regulation of morphogenesis of an epithelium	0.0718063492663983	3	37

EXCLI Journal 2024;23:1375-1396 – ISSN 1611-2156
Received: August 08, 2024, accepted: November 06, 2024, published: November 19, 2024

positive regulation of hormone metabolic process	0.0718063492663983	2	10
lipid transport	0.07707685791838295	7	265
nucleotide-binding domain, leucine rich repeat containing receptor signaling pathway	0.07707685791838295	3	38
regulation of CD4-positive, alpha-beta T cell differentiation	0.07707685791838295	3	38
organic hydroxy compound transport	0.07873851555563811	6	197
endothelial cell differentiation	0.07902453435092179	4	81
negative regulation of cell migration	0.07940640890950298	5	135
regulation of immunoglobulin production	0.08247629160615123	3	39
JNK cascade	0.08434359481346432	5	137
immunoglobulin secretion	0.08451395134268223	2	11
negative regulation of endothelial cell proliferation	0.08451395134268223	2	11
leukocyte mediated cytotoxicity	0.08451395134268223	4	83
plasminogen activation	0.08451395134268223	2	11
platelet alpha granule	0.08451395134268223	4	83
regulation of epithelial cell differentiation involved in kidney development	0.08451395134268223	2	11
microvillus	0.08451395134268223	2	11
positive regulation of blood coagulation	0.08451395134268223	2	11
regulation of plasminogen activation	0.08451395134268223	2	11
metanephric tubule development	0.08451395134268223	2	11
cell-substrate adhesion	0.08455021343831234	7	271
extrinsic component of plasma membrane	0.08515606755915292	5	138
negative regulation of protein secretion	0.08616771762792903	3	40
vasoconstriction	0.08616771762792903	3	40
nitric oxide biosynthetic process	0.08616771762792903	3	40
cell killing	0.08723352464406821	5	139
reactive oxygen species biosynthetic process	0.09082904315760898	4	85
positive regulation of nervous system development	0.09246876760775406	5	141
lymphocyte apoptotic process	0.09640695285362307	3	42
regulation of glial cell migration	0.09640695285362307	2	12
positive regulation of lymphocyte apoptotic process	0.09640695285362307	2	12
positive regulation of alpha-beta T cell proliferation	0.09640695285362307	2	12
phospholipase C-activating G protein-coupled receptor signaling pathway	0.09640695285362307	3	42
negative regulation of gene silencing by miRNA	0.09640695285362307	2	12
regulation of extracellular matrix disassembly	0.09640695285362307	2	12

EXCLI Journal 2024;23:1375-1396 – ISSN 1611-2156
Received: August 08, 2024, accepted: November 06, 2024, published: November 19, 2024

positive regulation of myeloid leukocyte mediated immunity	0.09640695285362307	2	12
regulation of cold-induced thermogenesis	0.09640695285362307	5	143
positive regulation of leukocyte mediated cytotoxicity	0.09640695285362307	3	42
negative regulation of blood vessel diameter	0.09640695285362307	3	42
cellular response to ketone	0.09640695285362307	3	42
renal vesicle development	0.09640695285362307	2	12
sterol transport	0.09640695285362307	4	87
positive regulation of steroid biosynthetic process	0.09640695285362307	2	12
negative regulation of tissue remodeling	0.09640695285362307	2	12
cellular response to fatty acid	0.09640695285362307	2	12
cytokine biosynthetic process	0.09640695285362307	2	12
cold-induced thermogenesis	0.09792684080195908	5	144
response to type I interferon	0.0990866365545073	4	88
cellular response to type I interferon	0.0990866365545073	4	88
extrinsic component of membrane	0.09998789911218108	6	210

EXCLI Journal 2024;23:1375-1396 – ISSN 1611-2156
Received: August 08, 2024, accepted: November 06, 2024, published: November 19, 2024

Supplementary Table 7: Possibile of base pairing of *FER1L4* pseudogene with miRNAs based on data from ENCORI

<table>
<tr><th>miRNA name</th><th>Gene name</th><th>Start</th><th>End</th><th>merClass</th><th>miRseq</th><th>Align</th><th>Target Sequence</th></tr>
<tr><td>hsa-miR-574-3p</td><td>FER1L4</td><td>34147999</td><td>34148021</td><td>7mer-m8</td><td>acacCCACACAC-GUACUCGCAc</td><td>|| | | | :|||||||</td><td>ccacGGAGCGGGAGGUGAGCGUc</td></tr>
<tr><td>hsa-miR-1306-5p</td><td>FER1L4</td><td>34148004</td><td>34148026</td><td>8mer</td><td>accugcaaaCGUC-CCCUCCACc</td><td>| || ||||||||</td><td>ccugcccacGGAGCGGGAGGUGa</td></tr>
<tr><td>hsa-miR-3605-3p</td><td>FER1L4</td><td>34148013</td><td>34148035</td><td>7mer-m8</td><td>gaucuccuguccauuGUGCCUCc</td><td>|||||||</td><td>cuuugacuaccugccCACGGAGc</td></tr>
<tr><td>hsa-miR-196a-5p</td><td>FER1L4</td><td>34148023</td><td>34148044</td><td>7mer-m8</td><td>ggguuguuguacuUUGAUGGAu</td><td>:|||||||</td><td>uguguuccgcuuuGACUACCUg</td></tr>
<tr><td>hsa-miR-196b-5p</td><td>FER1L4</td><td>34148023</td><td>34148044</td><td>7mer-m8</td><td>ggguuguuguccuUUGAUGGAu</td><td>:|||||||</td><td>uguguuccgcuuuGACUACCUg</td></tr>
<tr><td>hsa-miR-488-3p</td><td>FER1L4</td><td>34170794</td><td>34170814</td><td>8mer</td><td>cugguucuuuauCGGAAAGUu</td><td>||||||||</td><td>gagcucauugccGCCUUUCAa</td></tr>
<tr><td>hsa-miR-556-5p</td><td>FER1L4</td><td>34170806</td><td>34170827</td><td>7mer-m8</td><td>gaguauaauGUUACUCGAGUAg</td><td>|:: ||||||||</td><td>cugggcagcCGGAGAGCUCAUu</td></tr>
<tr><td>hsa-miR-670-3p</td><td>FER1L4</td><td>34170833</td><td>34170853</td><td>7mer-m8</td><td>aggacuuacuuauACUCCUUu</td><td>|||||||</td><td>caguucuucccccUGAGGAAg</td></tr>
<tr><td>hsa-miR-625-5p</td><td>FER1L4</td><td>34170840</td><td>34170861</td><td>7mer-m8</td><td>ccugauAUCUUGA-AAGGGGGa</td><td>|: |::| |||||||</td><td>cagaguUGCAGUUCUUCCCCCu</td></tr>
<tr><td>hsa-miR-514a-5p</td><td>FER1L4</td><td>34170855</td><td>34170876</td><td>7mer-m8</td><td>guacuaacagUGAGAGGUCUCAu</td><td>|| | |||||||</td><td>acccauaccaACGC-CCAGAGUu</td></tr>
<tr><td>hsa-miR-326</td><td>FER1L4</td><td>34170856</td><td>34170875</td><td>7mer-m8</td><td>gaccuccuucccCGGGUCUCc</td><td>||||||||</td><td>cccauaccaacGCCCAGAGu</td></tr>
<tr><td>hsa-miR-330-5p</td><td>FER1L4</td><td>34170856</td><td>34170877</td><td>7mer-m8</td><td>cggauUCUGUGUCCGGGUCUCu</td><td>| || | ||||||||</td><td>gacccAUACCAACGCCCAGAGu</td></tr>
<tr><td>hsa-miR-3196</td><td>FER1L4</td><td>34170896</td><td>34170913</td><td>8mer</td><td>cuCCGGGGACGGCGGGGc</td><td>||| |: ||||||||</td><td>agGGCACUGGCCGCCCCa</td></tr>
<tr><td>hsa-miR-6816-5p</td><td>FER1L4</td><td>34170896</td><td>34170915</td><td>8mer</td><td>cgUCCCUGGACGGGGCGGGGu</td><td>|||| ||| |||||||</td><td>gcAGGGCACUG-GCCGCCCCa</td></tr>
<tr><td>hsa-miR-3180</td><td>FER1L4</td><td>34170896</td><td>34170916</td><td>8mer</td><td>gaGGCCUUCGA--GGCGGGGu</td><td>| ||: || |||||||</td><td>ggCAGGGCACUGGCCGCCCCa</td></tr>
<tr><td>hsa-miR-3180-3p</td><td>FER1L4</td><td>34170896</td><td>34170919</td><td>8mer</td><td>ccggaGGCCUUCGA--GGCGGGGu</td><td>| ||: || |||||||</td><td>cugggCAGGGCACUGGCCGCCCCa</td></tr>
<tr><td>hsa-miR-874-3p</td><td>FER1L4</td><td>34170907</td><td>34170926</td><td>7mer-m8</td><td>agcCAGGGAGCCCGGUCCCGUc</td><td>||:||| ||| |||||||</td><td>cguGUUCCU-GGG-CAGGGCAc</td></tr>
<tr><td>hsa-miR-3173-5p</td><td>FER1L4</td><td>34170908</td><td>34170929</td><td>8mer</td><td>uuuccucuuuuGUCCGUCCCGu</td><td>::||||||||</td><td>ccccguguuccUGGGCAGGGCa</td></tr>
<tr><td>hsa-miR-6799-3p</td><td>FER1L4</td><td>34170908</td><td>34170930</td><td>8mer</td><td>gacacCCCUGUGGUACGUCCCGu</td><td>| | : |:: |||||||</td><td>cccccGUGUUCCUGGGCAGGGCa</td></tr>
<tr><td>hsa-miR-526b-5p</td><td>FER1L4</td><td>34191719</td><td>34191741</td><td>7mer-m8</td><td>ugucuuucacgaaGGGAGUUCUc</td><td>|||||||||</td><td>guuauguucagccCCCUCAAGAg</td></tr>
<tr><td>hsa-miR-140-3p</td><td>FER1L4</td><td>34195043</td><td>34195063</td><td>7mer-m8</td><td>ggcaccaagAUGGGACACCAu</td><td>| | |||||||</td><td>caagaaaaaUUCACUGUGGUc</td></tr>
<tr><td>hsa-miR-128-3p</td><td>FER1L4</td><td>34195045</td><td>34195065</td><td>7mer-m8</td><td>uuucUCUGGCCAAGUGACACu</td><td>||| |||||||||</td><td>aacaAGAAAAAUUCACUGUGg</td></tr>
<tr><td>hsa-miR-3681-3p</td><td>FER1L4</td><td>34195045</td><td>34195067</td><td>7mer-m8</td><td>ucaucacCUACUU-CGUGACACa</td><td>|| || |||||||</td><td>aaaacaaGAAAAAUUCACUGUGg</td></tr>
<tr><td>hsa-miR-216a-3p</td><td>FER1L4</td><td>34195045</td><td>34195069</td><td>7mer-m8</td><td>uaUUAGGGUCUCU---GGUGACACu</td><td>|| | ||| | :|||||||</td><td>agAAAACAAGAAAAAUUCACUGUGg</td></tr>
</table>

EXCLI Journal 2024;23:1375-1396 – ISSN 1611-2156
Received: August 08, 2024, accepted: November 06, 2024, published: November 19, 2024

Supplementary Table 8: Predicted mRNA targets regulated by miR-514a-5p, miR-330-5p and miR-128-3p

hsa-miR-514a-5p		
**Gene name**	**R-coefficient**	**p-value**
cldn16	-0.179	0.0001420000000
hla-dma	-0.134	0.0045100000000
kcna5	-0.101	0.0329000000000
kcnc1	-0.137	0.0036300000000
kcnk10	-0.224	0.0000016500000
lif	-0.326	0.0000000000014
rab33b	-0.095	0.0452000000000
ric3	-0.127	0.0070100000000
sash3	-0.198	0.0000245000000
sh3bgrl2	-0.138	0.0034400000000
tnfsf15	-0.141	0.0027100000000
**hsa-miR-330-5p**		
**Gene name**	**R-coefficeient**	**p-value**
adamts6	-0.116	0.013700000000
astn1	-0.104	0.027100000000
btn2a1	-0.142	0.002490000000
cbx7	-0.101	0.031700000000
crim1	-0.23	0.000000871000
kcnc1	-0.121	0.010300000000
kiaa0895	-0.123	0.009090000000
pcdh9	-0.265	0.000000011500
rcan2	-0.249	0.000000094100
rit1	-0.114	0.015400000000
slit2	-0.182	0.000109000000
spry2	-0.242	0.000000202000
wnt5a	-0.116	0.014300000000

hsa-miR-128-3p		
**Gene name**	**R-coefficeient**	**p-value**
abca1	-0.149	0.00156000000000
arl15	-0.143	0.00246000000000
bcl3	-0.101	0.03240000000000
btg2	-0.165	0.00044500000000
diras2	-0.12	0.01120000000000
ephb2	-0.117	0.01330000000000
f3	-0.142	0.00259000000000
fam13b	-0.12	0.01080000000000
fcrl2	-0.108	0.02170000000000
id2	-0.181	0.00011200000000
ikzf1	-0.111	0.01920000000000
ikzf2	-0.1	0.03400000000000
lpar6	-0.17	0.00028600000000
nap1l2	-0.15	0.00139000000000
nr1d2	-0.1	0.03380000000000
ntrk3	-0.121	0.01060000000000
plagl1	-0.116	0.01400000000000
ptger4	-0.142	0.00251000000000
ric3	-0.183	0.00009300000000
samd9l	-0.105	0.02570000000000
sema4d	-0.132	0.00514000000000
syt1	-0.125	0.00808000000000
tbx3	-0.137	0.00353000000000
trim23	-0.099	0.03520000000000
ttc39b	-0.108	0.02200000000000
unkl	-0.166	0.00042500000000
vegfc	-0.121	0.01050000000000
vip	-0.113	0.01650000000000
zeb1	-0.148	0.00164000000000
